# A Mosaic Layered Halide-Perovskite Spin Glass: Mechanochemical Alloying of a Ferromagnet and a Paramagnet

**DOI:** 10.1021/acscentsci.6c00194

**Published:** 2026-06-24

**Authors:** Julian A. Vigil, Murray Skolnick, Clara Zwanziger, Jiayi Li, Damara Dayton, Michael F. Toney, Salvatore Torquato, Hemamala I. Karunadasa

**Affiliations:** † Department of Chemistry, Stanford University, Stanford, California 94305, United States; ‡ Department of Chemical Engineering, Stanford University, Stanford, California 94305, United States; § College of Chemistry, University of California, Berkeley, Berkeley, California 94720, United States; ∥ Department of Chemistry, Princeton University, Princeton, New Jersey 08544, United States; ⊥ Department of Chemical and Biological Engineering, University of Colorado Boulder, Boulder, Colorado 80309, United States; # Materials Science and Engineering, University of Colorado, Boulder, Boulder, Colorado 80303, United States; ∇ Renewable and Sustainable Energy Institute (RASEI), University of Colorado Boulder, Boulder, Colorado 80309, United States; ○ Princeton Materials Institute, Princeton University, Princeton, New Jersey 08544, United States; ◆ Department of Physics, Princeton University, Princeton, New Jersey 08544, United States; ◧ Program in Applied and Computational Mathematics, Princeton University, Princeton, New Jersey 08544, United States; ◨ Stanford Institute for Materials and Energy Sciences, SLAC National Accelerator Laboratory, Menlo Park, California 94025, United States

## Abstract

Pulverizing together a Cr^II^ perovskite, (BA)_2_Cr^II^Cl_4_, and a Cr^III^ double perovskite, (BA)_4_Ag^I^Cr^III^Cl_8_, at room temperature affords a new layered perovskite alloy incorporating three different metal ions in each layer: (BA)_8_(Ag^I^Cr^III^)­Cr^II^_2_Cl_16_ (BA = *n*-butylammonium). The magnetic ground state of this alloy is a spin glass (freezing temperature ∼ 3 K), which we propose arises from intrinsic disorder of superexchange interactions and a propensity to form ferromagnetic clusters. To explore the composition space beyond this example, we model the geometrical and topological properties of these complex alloys by representing the [MCl_6_]^*n*−^ tiling as effectively hard-rhombus packings on a square lattice. The structures provided by our computationally efficient model provide insight into magnetic exchange, ordering across various length scales, and the role of the in-plane Jahn–Teller distortion of the Cr^II^ centers in dictating the packing within the inorganic layer. By quantifying local compositional fluctuations, we identify alloy compositions at which the mixing characteristics are appreciably different from those observed in random square-lattice mixtures of three different metals. These results demonstrate a general, mechanochemical route to two-dimensional spin glasses and provide design principles and computational tools for expanding the phase space of complex layered halide perovskites with nontrivial magnetic ground states.

## Graphical abstract

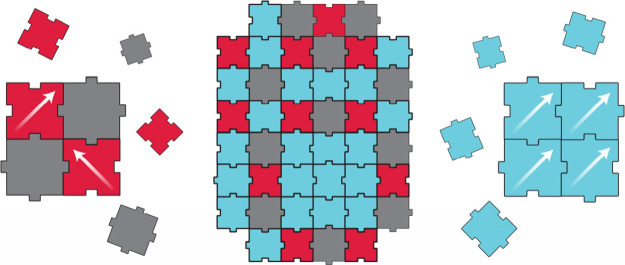

## Introduction

Layered or 2D halide perovskites—derived from dimensional reduction of the *A* ^I^ *B* ^II^ *X*_3_ 3D perovskites with corner-sharing metal-halide octahedra—exhibit distinctive optoelectronic properties^1,2^ owing to dielectric- and quantum-confinement effects.^3,4^ Foremost among these properties are (*i*) the bright, excitonic photoluminescence of 2D lead-halide perovskites with thin inorganic sheets, motivating applications in phosphors^5^ and light-emitting diodes,^6^ and (*ii*) the photocurrent of 2D perovskites with thicker inorganic sheets, which has led to their use in photovoltaics.^7^ Although less investigated, layered perovskites with transition metals are model systems for 2D magnetism. Indeed, layered fluoro-chromate and chloro-chromate perovskites were investigated extensively as transparent ferromagnets in the 1960s.^8–11^

Magnetic exchange interactions mediated through bridging halides in perovskites can be understood through the Goodenough-Kanamori-Anderson rules for magnetic coupling between linear *B*–*X*–*B* bonds,^12^ particularly when octahedral tilting is minimal. Superexchange between neighboring octahedral *B*-site metals in single perovskites is typically antiferromagnetic since the unpaired electrons reside in orbitals of the same symmetry (dictated by the Pauli exclusion principle), as seen in Fe^II^ and Mn^II^ perovskites.^13^ However, perovskites with *B*-site metals that feature a pronounced tetragonal distortion due to the Jahn–Teller effect show a distinctive tiling pattern. These perovskites crystallize with mutually perpendicular elongated metal–ligand axes lying along the 2D perovskite layer; this arrangement causes the unpaired electrons to reside in mutually orthogonal orbitals, leading to ferromagnetic coupling (dictated by Hund’s rules) in Cu^II^ (d^9^) and Cr^II^ (high-spin d^4^) perovskites.^13,14^ Thus, the strong intralayer ferromagnetic coupling in layered *A*_2_ *BX*_4_-type (*A* = monocation; *B* = Cu^II^, Cr^II^; X = F^–^, Cl^–^, Br^–^) perovskites overwhelms the weak antiferromagnetic interlayer interactions, yielding bulk ferromagnets with ordering temperatures (*T*_*C*_) as high as 90 K.^8–11,13,14^

To expand the compositional diversity of layered halide perovskites, we recently devised methods to incorporate stoichiometric 1+, 2+, and 3+ metals in the *B* site.^15,16^ The first example,^15^ *A*_8_(Cu^I^In^III^)­Cu^II^_2_Cl_16_, had crystallographically indistinguishable *B* sites, which could be modeled as 3:1 Cu:In mixed-occupancy sites. This composition could be conceptually derived from a 2:1 alloy of a layered single perovskite (*A*_2_Cu^II^Cl_4_) and double perovskite (*A*_4_Cu^I^In^III^Cl_8_). Unlike most alloys that form a solid solution over a wide range of mixing ratios, however, deviations from this 2:1 precursor ratio only led to a decrease in yield—rather than crystallization of other perovskite compositions—which led us to propose a tiling model that enforces a Cu^I^:In^III^:Cu^II^ ratio of 1:1:2. This model requires that the long axis of the Cu^II^ center bridges to the smaller In^III^ center and that the short axis of the Cu^II^ center bridges to the larger Cu^I^ center. We refer to these alloys as “mosaic perovskites” due to the various distinct local tiling arrangements that can preserve this overall stoichiometry.

In an uncorrelated *B*-site mixture, the probability that any *B* site is occupied by a given metal is equal to its mole fraction. The in-plane elongation of the Cu^II^ center appears to dictate the mosaic tiling of the *B*-site metals to minimize packing mismatches; thus, these alloys are not random/uncorrelated *B*-site mixtures and are expected to display varying degrees of miscibility and order across length scales. The Cu^I^–Cu^II^ mosaic perovskites^15,16^ are just the second example of mixed-valence 2D halide perovskites,^17–21^ and they exhibit emergent optoelectronic properties due to intervalence Cu^I^→Cu^II^ charge transfer. However, the dilution of the paramagnetic Cu^II^ centers, with diamagnetic Cu^I^ and In^III^, to below the concentration needed to support sample-spanning ferromagnetic domains^22–26^ led to the loss of ferromagnetic order. Thus, the mosaic perovskite was a simple paramagnet,^15^ motivating us to extend the mosaic compositions to include two paramagnetic ions and access more complex magnetic behavior.

Herein we report a new mixed-valence chlorochromate mosaic perovskite. This mosaic Cr^II^–Cr^III^ layered perovskite is synthesized from a Cr^II^ single-perovskite ferromagnet and a Ag^I^–Cr^III^ double-perovskite paramagnet using simple mechanochemical milling at room temperature. Unlike the Cu^I^–Cu^II^ perovskites, the Cr^II^–Cr^III^ perovskites form across a range of compositions and support sample-spanning domains of paramagnetic *B*-site ions. Here, we experimentally investigate the Ag^I^:Cr^III^:Cr^II^ 1:1:2 composition; others will be reported in a future publication.

To model the local tiling patterns of the *B*-site octahedra within a single layer, we adapt the computationally efficient Adaptive Shrinking Cell (ASC) stochastic algorithm^27–30^ —an established hard-particle packing protocol for particles in Euclidean space—to the case of impenetrable rhombi (i.e., the planar cross sections of the metal-halide octahedra) fixed to a square lattice. We call this adaptation the Lattice Adaptive Shrinking Cell (LASC) algorithm and use LASC simulations to generate large (200^2^ rhombi) arrangements of the square-lattice-bound *B*-site octahedra subject to the constraint of minimizing packing mismatch between the shared vertices of nearest-neighbor octahedra. This algorithm was recently shown to simulate the proposed tiling of *A*_8_(Cu^I^In^III^)­Cu^II^_2_Cl_16_ that agreed with experimental observations.^31^

Leveraging the high-resolution structural snapshots provided by our LASC simulations, we find that the Cr^II^–Cr^III^ alloys exhibit more complex composition-dependent order at the *B* sites. We recently established that both the degree of disorder^32^ and mixing^33^ in heterogeneous materials exhibit nontrivial dependence on length scale using novel metrics that quantify local compositional fluctuations. Here, we apply these analytical tools to individual rhombi in the LASC structures and identify significant differences between the collective arrangements of the mosaic alloys and random (uncorrelated) *B*-site mixtures of three different metals. Finally, we experimentally show that the magnetic ground state of the (BA)_8_(Ag^I^Cr^III^)­Cr^II^_2_Cl_16_ (BA = *n*-butylammonium) alloy is a spin glass. This complex ground state arises from a mix of superexchange interactions and a propensity to form stable ferromagnetic clusters—consistent with our LASC modeling of mixing and topological connectedness in these alloys.

## Results

### The Layered Chlorochromate Precursor Perovskites

Similar to the syntheses of other *A*_2_Cr*X*_4_ perovskites (*A* = organoammonium; X = F^–^, Cl^–^, Br^–^),^8–11^ the Cr^II^ perovskite (BA)_2_CrCl_4_ crystallizes as colorless plates from solution in inert atmosphere (see Methods). The tetragonal distortion of the Cr^II^ centers was originally deduced from the interpretation of optical absorption spectra, although the Jahn–Teller distortion was not structurally resolved until X-ray diffraction studies of K_2_CrCl_4_.^14^ Here, we solve the structure of (BA)_2_CrCl_4_ from analysis of single-crystal and powder X-ray diffraction (SCXRD and PXRD, respectively) measurements (see Methods and Supporting Information, Figure S1 and Table S1); the structure solution shows the tetragonal distortion arising from high-spin Cr^II^ and the in-plane orthogonal arrangement of nearest-neighbor elongated Cr^II^–Cl bonds (Figure 1A). The unique Cr^II^–Cl bond lengths are 2.37 Å (*ax*), 2.40 Å (*eq*), and 2.84 Å (*eq*).

**Figure 1:**
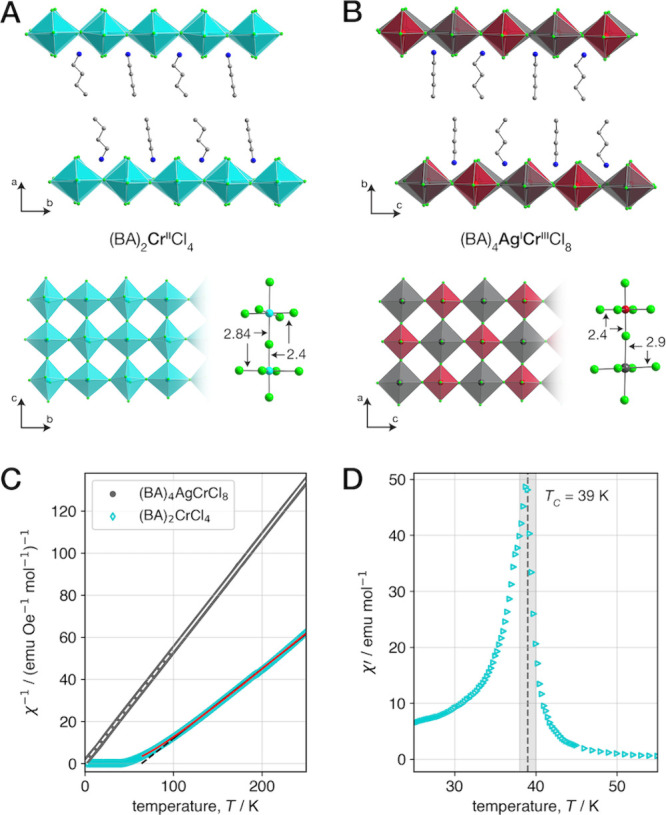
**Structure and bulk magnetic properties of the layered Cr** ^**II**^ **single and Ag** ^**I**^ **–Cr** ^**III**^ **double perovskites**. Structure solutions of (BA)_2_CrCl_4_ (**A**) and of (BA)_4_AgCrCl_8_ (**B**) from refinement of powder X-ray diffraction data (see main text for details); the intralayer order and equatorial bonding (bond lengths in Å) within each perovskite is highlighted in plane view (bottom). Turquoise, gray, and crimson polyhedra represent [Cr^II^Cl_6_], [Ag^I^Cl_6_], and [Cr^III^Cl_6_], respectively, and green, blue, and gray spheres represent Cl, N, and C atoms, respectively. BA = *n*-butylammonium; disorder and H atoms omitted for clarity. (**C**) Inverse DC susceptibility (χ^–1^) from SQUID magnetometry measurements of (BA)_2_CrCl_4_ (turquoise) and (BA)_4_AgCrCl_8_ (gray) powders, including corresponding Curie–Weiss (black/white lines) and exchange-constant fits (red line; see main text); solid lines represent the fitted region in temperature, and dotted lines are the extrapolation to the Curie–Weiss temperature at χ ^–1^ = 0 for visual reference. (**D**) Zero-field AC susceptibility from SQUID magnetometry measurements of (BA)_2_CrCl_4_ (with a frequency of 757 Hz and drive field of 1 Oe) near the bulk ordering temperature (*T*_*C*_, dotted vertical line).

The new Cr^III^ layered double perovskite (BA)_4_AgCrCl_8_ was synthesized from the solid precursors in an evacuated glass ampule, similar to approaches employed previously for other layered chloride double perovskites.^34^ Phase-pure purple powders were obtained after two grind-heat cycles (see Methods); the polycrystalline product of these solid-state reactions exhibited intergrown domains, precluding SCXRD analysis. We therefore employed single-crystal electron diffraction (SCED, or microED) for space-group determination and high-resolution synchrotron PXRD to refine the structure (see Methods and Supporting Information, Figure S2 and Table S2). The structure solution shows characteristic rock-salt ordering of alternating Ag^I^ and Cr^III^ sites (Figure 1B) and approximately undistorted octahedral coordination of Cr^III^ with equatorial Cr^III^–Cl bond lengths of 2.40 Å.

The bulk magnetic properties of (BA)_2_CrCl_4_ and (BA)_4_AgCrCl_8_ were measured from polycrystalline powders using a SQUID magnetometer (see Methods). Variable-temperature direct-current (DC) magnetic susceptibility (χ) data were corrected for diamagnetic contributions and modeled using the Curie–Weiss (CW) law, χ = *C*/(*T* – *θ*_*CW*_), where *T* is temperature, *C* is the Curie constant, and *θ*_*CW*_ is the CW temperature. The inverse susceptibility (χ^–1^) data were fit to the CW law for 100 K < *T* < 250 K (Figure 1C); fitted parameters are given in Table S3 (Supporting Information). Inspection of χ^–1^ and of the CW fits indicate that (BA)_4_AgCrCl_8_ is a simple paramagnet with an effective moment (*μ*_*eff*_) corresponding to the spin-only moment of Cr^III^ (expected, 3.87*μ*_*B*_; fitted, 3.86*μ*_*B*_). Ferromagnetic coupling in (BA)_2_CrCl_4_ is evident from the positive *θ*_*CW*_ (64 K; Figure 1C). Variable-temperature zero-field alternating current (AC) magnetic susceptibility measurements (Figure 1D) allow for the bulk ferromagnetic ordering temperature (*T*_*C*_ = 39 K) to be determined from the maximum of the in-phase (χ′) susceptibility. Fitting χ^–1^ above *T*_*C*_ for (BA)_2_CrCl_4_ to a series expansion representing nearest-neighbor exchange coupling in a layered Heisenberg magnet^35,36^ (Figure 1C) yields estimates of the intralayer coupling constant *J* [0.8891(9) meV] and the *g*-factor [1.9411(5)], both in line with the magnetism of other layered tetrachlorochromate perovskites.^36,37^

### The Layered Cr^II^–Cr^III^ Mosaic Perovskites

Similar to the formation of Cu^I^–Cu^II^ mosaic perovskites,^15,16^ we posited that the Jahn–Teller distortion of high-spin Cr^II^ in (BA)_2_CrCl_4_ could facilitate the formation of a mosaic alloy via mechanochemical synthesis. The synthetic approach employed here to produce polycrystalline alloys is generalized in Scheme 1. Briefly, we combined stoichiometric mixtures of (BA)_2_CrCl_4_ and (BA)_4_AgCrCl_8_ powders with zirconia media in N_2_ atmosphere and milled the mixture for 2 h at 800 rotations per minute to afford a brown solid (see Methods and Scheme 1). The general formula of the product is (BA)_2(2*y*+*x*)_(Ag^I^Cr^III^)_*y*_Cr^II^_*x*_Cl_4(2*y*+*x*)_ [denoted **(Ag** ^**I**^ **Cr** ^**III**^ **)**_***y***_ **Cr** ^**II**^_***x***_ for simplicity]; we focus our attention in characterization on **(Ag** ^**I**^ **Cr** ^**III**^ **)­Cr** ^**II**^_**2**_ but return to discuss mixing more generally below. Elemental analysis (CHN and Ag:Cr) of **(Ag** ^**I**^ **Cr** ^**III**^ **)­Cr** ^**II**^_**2**_ was consistent with the empirical formula (C_4_H_12_N)_8_AgCr_3_Cl_16_ (see Methods). Retention of BA at the *A*-site in the layered perovskite structure is inferred from characteristic C–H, C–N, and N–H vibrational modes in the infrared spectrum (Figures S3–S4, Supporting Information).

**Scheme 1:**
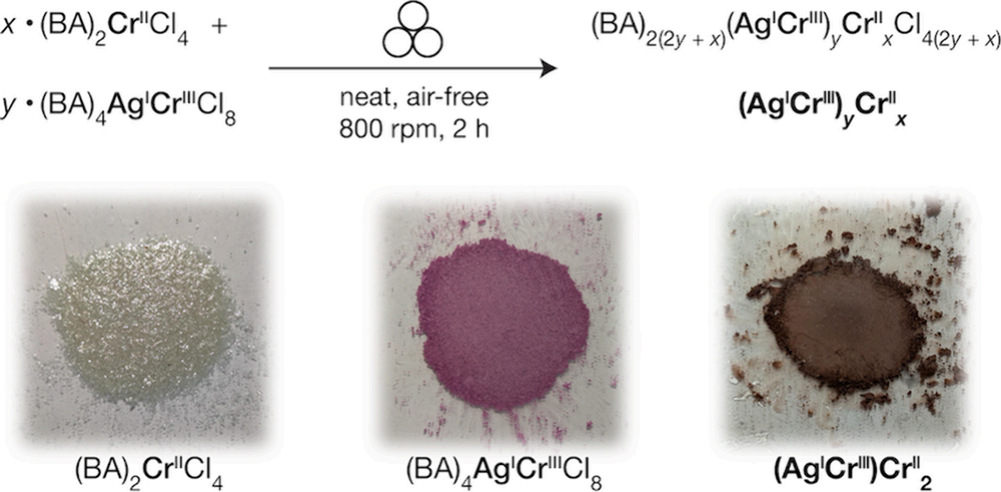
(Top) Solid-State Mechanochemical Synthesis of Chloride Perovskite Alloys.^a^ (Bottom) Representative Optical Photographs of Bulk Polycrystalline Powders of the Precursors and (BA)_8_AgCr_3_Cl_16_ [**(Ag** ^**I**^ **Cr** ^**III**^ **)­Cr** ^**II**^_**2**_]­^b^

a The general formula (BA)_2(2*y*+*x*)_(Ag^I^Cr^III^)_*y*_Cr^II^_*x*_Cl_4(2*y*+*x*)_ is denoted **(Ag** ^**I**^ **Cr** ^**III**^ **)**_***y***_ **Cr** ^**II**^_***x***_; BA = *n*-butylammonium.
b Powders were pressed between borosilicate glass microscope slides and photographed through the panel of a N_2_-filled glovebox.

The **(Ag** ^**I**^ **Cr** ^**III**^ **)­Cr** ^**II**^_**2**_ alloy is expected to adopt the layered perovskite structure with a disordered *B*-site.^15^ Indeed, the positions of the Bragg reflections from PXRD measurements of **(Ag** ^**I**^ **Cr** ^**III**^ **)­Cr** ^**II**^_**2**_ coincide with those of the precursors but exhibit considerable broadening (Figure 2A), prohibiting determination of the symmetry of the average structure. A shift in the (*h*00) reflections toward smaller 2θ values indicate expansion normal to the inorganic layers upon formation of the mosaic alloy. High-resolution neutron powder diffraction measurements (*T* = 100 K; see Methods) corroborate this observation, showing an increase in the largest average interplanar spacing (*d*) from 14.09 Å and 14.22 Å in the precursors [(BA)_4_AgCrCl_8_ and (BA)_2_CrCl_4_, respectively] to 14.64 Å in **(Ag** ^**I**^ **Cr** ^**III**^ **)­Cr** ^**II**^_**2**_ (Figure 2B). The breadth of the Bragg reflections from **(Ag** ^**I**^ **Cr** ^**III**^ **)­Cr** ^**II**^_**2**_ further reflects a distribution of *d* values, likely originating from heterogeneity or small domain sizes in the mechanochemically derived alloy.

**Figure 2:**
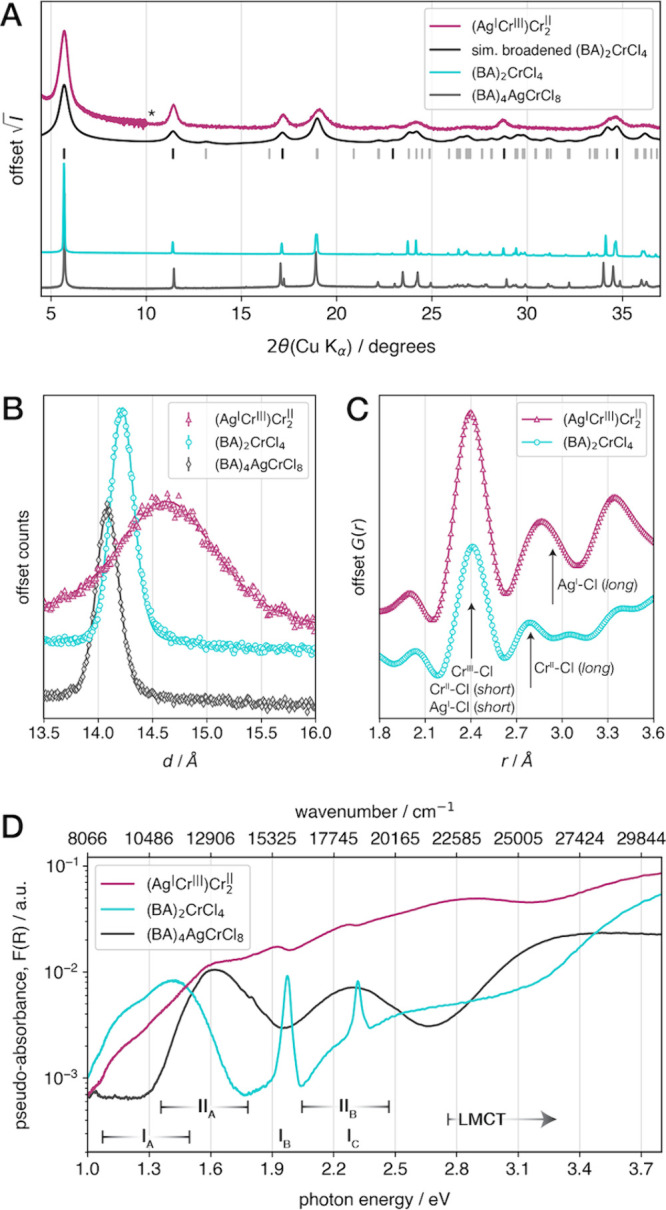
**Characterization of the (Ag** ^**I**^ **Cr** ^**III**^ **)­Cr** ^**II**^_**2**_ **mosaic perovskite**. (**A**) Powder X-ray diffraction patterns of the precursor single and double perovskites [(BA)_2_CrCl_4_ and (BA)_4_AgCrCl_8_, respectively] and the **(Ag** ^**I**^ **Cr** ^**III**^ **)­Cr** ^**II**^_**2**_ mosaic perovskite; allowed Bragg reflections and a simulated pattern from the structure solution of (BA)_2_CrCl_4_ are shown for comparison [bolded tick marks are associated with the largest interplanar spacing, e.g., (*h*00)]; the asterisk denotes a change in measurement parameters for **(Ag** ^**I**^ **Cr** ^**III**^ **)­Cr** ^**II**^_**2**_. (**B**) High-*d* powder neutron diffraction patterns of (BA)_2_CrCl_4_, (BA)_4_AgCrCl_8_, and **(Ag** ^**I**^ **Cr** ^**III**^ **)­Cr** ^**II**^_**2**_ (symbols), measured at *T* = 100 K; average interplanar spacing (*d*) was determined from a pseudo-Voigt fit (solid lines). (**C**) X-ray pair distribution function from total scattering measurements of (BA)_2_CrCl_4_ and **(Ag** ^**I**^ **Cr** ^**III**^ **)­Cr** ^**II**^_**2**_. (**D**) Room-temperature diffuse reflectance UV–vis spectra of (BA)_2_CrCl_4_, (BA)_4_AgCrCl_8_, and **(Ag** ^**I**^ **Cr** ^**III**^ **)­Cr** ^**II**^_**2**_; *d-d* transition assignments are denoted with roman numerals; I for (BA)_2_CrCl_4_ and II for (BA)_4_AgCrCl_8_. Ligand-to-metal charge transfer assignments are denoted LMCT; pseudoabsorbance was calculated from the measured reflectance, *R*, using the Kubelka–Munk transformation.^40^

We employed pair distribution function (PDF) analysis from X-ray total scattering measurements to investigate interatomic distances in the alloy (see Methods). The first coordination sphere of the *B*-site metals shows a large peak in *G*(*r*)—for both the precursor (BA)_2_CrCl_4_ and **(Ag** ^**I**^ **Cr** ^**III**^ **)­Cr** ^**II**^_**2**_—centered on 2.4 Å (Figure 2C), consistent with short Cr^II^–Cl bonds, the Cr^III^–Cl bond, as well as the axial Ag^I^–Cl bond. Most notable is the peak centered on 2.8 Å for (BA)_2_CrCl_4_, originating from the elongated Cr^II^–Cl bond (Figure 2C); upon alloy formation, this feature is maintained in the *G*(*r*) for **(Ag** ^**I**^ **Cr** ^**III**^ **)­Cr** ^**II**^_**2**_ and combines with the equatorial Ag^I^–Cl bond (ca. 2.9 Å) to produce a broad peak extending above 3 Å.

Diffuse-reflectance ultraviolet–visible (DR-UV–vis) spectra of the precursor perovskites and the **(Ag** ^**I**^ **Cr** ^**III**^ **)­Cr** ^**II**^_**2**_ alloy are shown in Figure 2D; (BA)_2_CrCl_4_ and **(Ag** ^**I**^ **Cr** ^**III**^ **)­Cr** ^**II**^_**2**_ were measured in an air-free holder to avoid oxidation of Cr^II^ in ambient atmosphere (see Methods for details). Electronic excitations in the UV–vis region for the chlorochromates are attributed to *d*-*d* transitions for energies below 2.8 eV; at higher energies, the absorption is dominated by ligand-to-metal (Cl→Cr) charge transfer transitions (LMCT, Figure 2D). The double perovskite (BA)_4_AgCrCl_8_ exhibits two broad, spin-allowed *d*-*d* transitions centered on 1.6 and 2.3 eV (II_A_ and II_B_, Figure 2D). The distinct features arise from the ^4^A_2g_→^4^T_2g_ and ^4^A_2g_→^4^T_1g_ transitions, respectively, and are thought to broaden (or split) through lattice vibrations and trigonal distortions from octahedral coordination in the chlorochromates.^38,39^

Exemplified by the optical spectra of the single perovskite (BA)_2_CrCl_4_, the *d*-*d* transitions of high-spin Cr^II^ are particularly rich in information regarding order and spin dynamics. A wide envelope of intensity between 0.9 eV (instrument cutoff) and 1.8 eV is assigned to the spin-allowed *d*-*d* transition from the quintet ground state (I_A_, Figure 2D), where the linewidth is also broad due to vibrations and distortions. Two sharp transitions are observed at ca. 1.95 and 2.33 eV (I_B_ and I_C_, Figure 2D). These quintet-to-triplet transitions have been regarded as a remarkable feature of the optical properties of *A*_2_CrCl_4_-type ferromagnets;^8–11,14^ the intensity of these formally spin-forbidden transitions increases quadratically with temperature (up to *T*_C_) and often persists at room temperature.

The optical cross section is rationalized by invoking momentum-conserving magnon-exciton coupling.^36,41^ To offset the reduction in spin angular momentum from the optical spin-flip transition, a corresponding increase in angular momentum originates from the annihilation of a thermally excited magnon.^36,37,41^ The diminished intensity of transitions I_B_ and I_C_ for the **(Ag** ^**I**^ **Cr** ^**III**^ **)­Cr** ^**II**^_**2**_ alloy (Figure 2D) is consistent with the loss of long-range ferromagnetic ordering and distinct magnon dynamics, concomitant with Cr^II^ dilution and *B*-site disorder. Ball-milling (BA)_2_CrCl_4_ alone also leads to a reduction in the intensity of the spin-forbidden transitions (Figure S5, Supporting Information) while the structure is maintained (as determined by PXRD; Figure S6, Supporting Information), which may be due to decreased magnon lifetimes upon reducing the crystal size from macroscopic to micrometer-scale powders (e.g., through increased surface-impurity-induced scattering^42^). Thus, we cannot quantitatively connect changes in the spin-forbidden transitions to the magnetization of **(Ag** ^**I**^ **Cr** ^**III**^ **)­Cr** ^**II**^_**2**_.

### Simulating Mosaic Alloys across Various Length Scales

A schematic representation of a possible 1:2 mixture [*mol* (BA)_4_AgCrCl_8_: *mol* (BA)_2_CrCl_4_] of double- and single-perovskite *B*–Cl units is shown in Figure 3A, where the units are planar and effectively “rigid” (up to small degrees of overlaps as detailed below). A bridge between the short axis of the Cr^II^–Cl rhombus and the axes of the larger Ag^I^–Cl rhombus produces a hypothetical structure with minimal mismatch between neighbors. In qualitative comparisons, rhombus axis lengths are defined along the bonds and thus correspond to Cl–*M*–Cl length(s). We posit that small overlaps and gaps (i.e., 1–2% of the rhombus axes^15^) are comparable to atomic displacements arising from normal vibrational modes and incoherent thermal motion. In contrast, larger vertex–vertex mismatch (>5% of the rhombus axes) of nearest-neighbor metal-chloride rhombi would likely cause unphysical bond lengths or large out-of-plane octahedral tilting.

**Figure 3:**
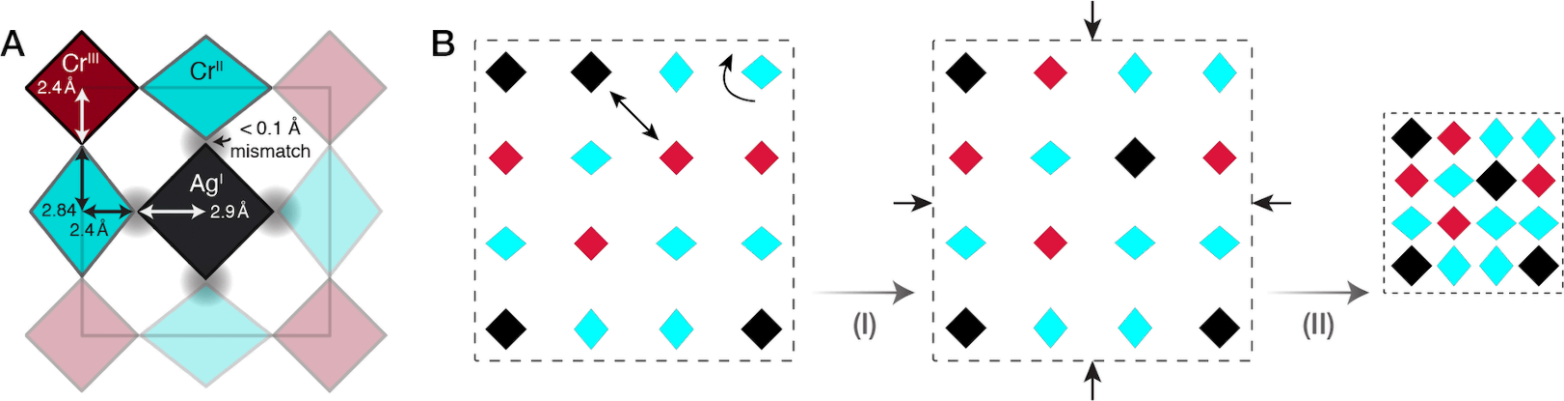
**In-plane geometric packing and simulation of effectively rigid metal-chloride rhombi**. (**A**) Schematic representation of a tile in a hypothetical 1:2 [*mol* (BA)_4_AgCrCl_8_: *mol* (BA)_2_CrCl_4_] mixture of single- and double-perovskite units with minimal mismatch. Tessellation and rotation of this tile afford the disordered mosaic perovskite with fixed stoichiometry. (**B**) Schematic representation of the trial Monte Carlo swap and rotational (I) and isotropic cell compression (II) moves employed by the LASC algorithm, as well as the resulting densified system. In a dilute initial configuration, a Ag^I^ and a Cr^III^ rhombus are randomly selected for a trial swap move, while a Cr^II^ rhombus in the top-right corner is randomly selected for a trial rotational move (I); later, the trial moves are accepted and an isotropic compression is applied to the simulation cell (II; the periodic boundary of the cell is denoted by the black dashed square); finally, the trial compression is accepted, resulting in a more closely packed arrangement. Note that the magnitude of the compression is greatly exaggerated in the figure for clarity. Adapted with permission from ref 31. Copyright 2025 AIP Publishing.

Given the geometrical packing constraints in the formation of these alloys, we employ an adaptation of the ASC scheme^27–30^ for lattice packings, called the LASC scheme, to generate candidate arrangements of *B*-site rhombi with minimal vertex–vertex mismatch.^31^ In LASC, the *B*–Cl (*B* = Ag^I^, Cr^II^, Cr^III^) units are represented as effectively rigid rhombi that are fixed to a square lattice with lattice constant *a* at the prescribed stoichiometry and interact through vertex–vertex interactions via the pair potential:^31^

1 $$v\left(r_{ij}\right)=k\left({\left|\frac{r_{ij}}{\sigma }\right|}^{\alpha }+{\left|\frac{r_{ij}}{\sigma }\right|}^{\beta }\right)$$

in which *r*_*ij*_ is the distance (in Å) between the adjacent vertices of the nearest-neighbor rhombi at sites *i* and *j*, the parameters σ > 0 and *k* > 0 respectively control the characteristic length scale and strength of the interaction, and the exponents α and β are described below. The vertex–vertex interaction potential (eq 1) relaxes the strict nonoverlapping constraint of the original ASC scheme by allowing overlaps (*r*_*ij*_ < 0) between neighboring rhombi; however, such overlaps remain penalized, effectively capturing hard-particle packing constraints. Unlike the case of true hard-particle systems, gaps (*r*_ij_ >0) between neighboring vertices are also penalized by the potential.

Small (i.e., |*r*_*ij*_| is much less than the rhombus axis length) overlaps and gaps are penalized by taking 0 < α < 1, whereas large (i.e., |*r*_ij_| is comparable to the rhombus axis length) gaps and overlaps are penalized by taking β ≥ 2. For simplicity, gaps and overlaps of equal magnitude |*r*_ij_| incur the same energetic penalty. The aforementioned ranges on the exponents were ascertained via trial and error using the crystal structures of the single and double perovskite precursors as benchmarks^31^ (see Figure S7, Supporting Information). Here, we specifically take α = 0.5 and β = 2 but note that other combinations (e.g., α = 0.1 and β = 4) should yield similar results. The length scale σ = 1 Å because the interactions captured by the potential (eq 1) occur over distances on the order of the bond length. The interaction strength *k* sets the energy scale of the system, so it is taken to be unity without loss of generality.

Our LASC model accurately captures nearest-neighbor vertex–vertex interactions, including static distortions (e.g., octahedral tilting), through the simple heuristic interaction potential (eq 1). Symmetry-lowering octahedral tilting is not explicitly considered in the LASC scheme given that a large vertex–vertex overlap (>5%) would correspond to a large out-of-plane tilt of the octahedra (*M*–*X*–*M* bond angle projection of ca. > 18°; see Figure S8, Supporting Information). Overall, the problem of finding dense arrangements of the rhombi with minimal packing mismatch amounts to finding minima of the fictitious energy:

2 $$E=\underset{\left\langle i,j\right\rangle }{\sum }v\left(r_{ij}\right)$$

in which ⟨*i*,*j*⟩ denotes summation over nearest-neighbor pairs. The LASC algorithm solves this minimization problem (eq 2) using simulated annealing: an initial randomized, low-density arrangement of the prescribed composition of 200 × 200 *B*-site rhombi is densified through a series of irreversible isotropic cell compression steps, as shown in Figure 3B. Random trial swap and discrete 90° rotational moves [Figure 3B, (I); acceptance is governed by the Metropolis criterion^43^] attempt to remove packing mismatches introduced by the compression steps [Figure 3B, (II)].

Specifically, by employing a minimalist, effective hard-particle packing model, the LASC simulations are computationally efficient, enabling the generation of a 200 × 200 *B*-site alloy structure in ca. 3.5 h on a single CPU core. Specific simulation parameters, LASC algorithm details, and benchmark simulation results are detailed in our prior report^31^ and provided in the Methods section and the Supporting Information. The LASC algorithm can be generalized to model the more complex case of 3D mosaic alloys as packings of cubic-lattice-bound effectively rigid octahedra. In this case, one would add an additional set of vertex–vertex interactions governed by eq 1 to capture the additional packing constraints present in 3D structures.^31^

We used the LASC algorithm to investigate the possibility of forming tunable disordered magnets by combining (BA)_2_CrCl_4_ and (BA)_4_AgCrCl_8_ at various ratios. The simulations predict nontrivial mixing across a range of compositions (Figure 4A and Figures S9–S10, Supporting Information), suggesting that various mixtures could be accessible by mechanochemical alloying. As the presence of simulation-cell-spanning clusters of paramagnetic ions suggests the possibility of bulk magnetic order,^15,24^ we estimated nearest-neighbor site percolation thresholds (i.e., the critical fraction of occupied lattice sites at which a simulation-cell-spanning cluster first appears) for two subsets of Cr networks: all Cr vs Cr^II^ only. Importantly, the percolation thresholds for both subsets of Cr networks in the LASC structures are dramatically different from those of the Bernoulli model (i.e., random-site nearest-neighbor percolation on the square lattice), which is 0.592.

We first consider percolating networks of *all* nearest-neighbor Cr ions at the *B*-site in the LASC structures. The right-hand side of Figure 4A highlights this network within a simulated **(Ag** ^**I**^ **Cr** ^**III**^ **)­Cr** ^**II**^_**2**_ LASC structure where isolated domains of endmember double perovskite ordering are not shown. The terms “endmember single perovskite” and “endmember double perovskite” (Figure 1A) refer to the *y* = 0 and *x* = 0 limits of the alloy **(Ag** ^**I**^ **Cr** ^**III**^ **)**_***y***_ **Cr** ^**II**^_***x***_, respectively. Intriguingly, Cr ions within a range of simulated **(Ag** ^**I**^ **Cr** ^**III**^ **)Cr** ^**II**^_***x***_ compositions (*x* ≥ 0.5; or *B*-site mole fraction of Cr^II^, *X*_*Cr(II)*_ ≥ 0.2) span the simulation cell in a continuous network, with 100% of the 250 *B*-site alloy configurations exhibiting percolating clusters of Cr (Figure 4B and Figure S10, Supporting Information). If we restrict Cr^II^–Cr^II^ interactions in the percolating network to nearest-neighbor units with orthogonal elongated axes (i.e., endmember single perovskite ordering, Figure 1A), we still observe that 100% of the **(Ag** ^**I**^ **Cr** ^**III**^ **)­Cr** ^**II**^_***x***_ structures exhibit percolating clusters of Cr ions for *x* ≥ 1 (Figure 4B). We therefore estimate the percolation threshold of paramagnetic Cr ions in these **(Ag** ^**I**^ **Cr** ^**III**^ **)**_***y***_ **Cr** ^**II**^_***x***_ alloys to be *X*_*Cr*_ ∼ 0.67 (*x* = *y* = 1). The critical mole fraction of Cr^II^ associated with the phase transition—from disconnected magnetic domains to simulation-cell-spanning clusters—is thus within the range 0.2 < *X*_*Cr(II)*_ < 0.33 [0.5 < *x* < 1 in **(Ag** ^**I**^ **Cr** ^**III**^ **)­Cr** ^**II**^_***x***_], depending on the constraints placed on nearest-neighbor Cr^II^ interactions.

**Figure 4:**
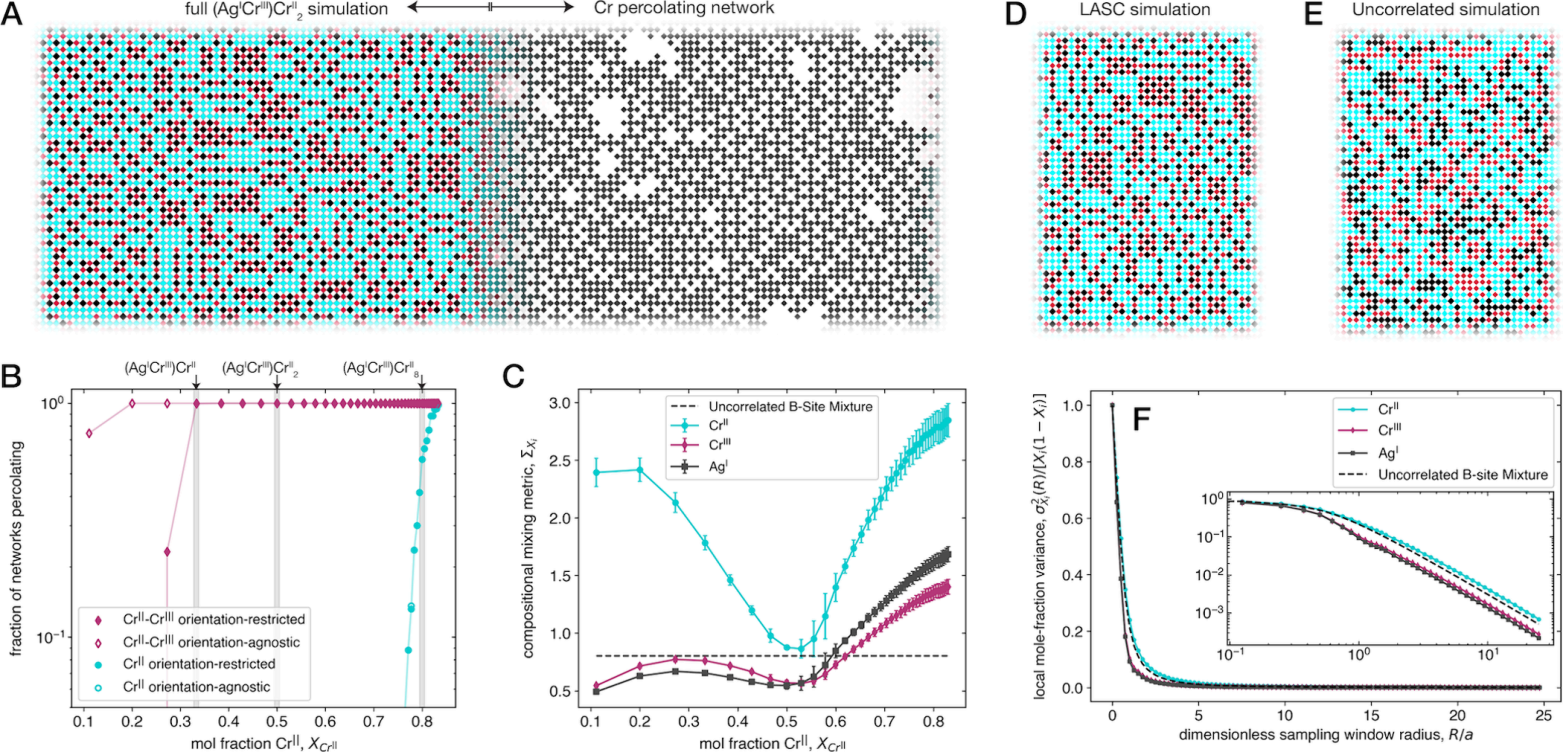
**Large-cell LASC simulation of the mosaic perovskites**. (**A**) Left: Section of a representative large-cell LASC simulation of an alloy of metal-chloride rhombi, with the stoichiometry of the **(Ag** ^**I**^ **Cr** ^**III**^ **)­Cr** ^**II**^_**2**_ mosaic perovskite; Ag^I^–Cl, Cr^III^–Cl, and Cr^II^–Cl rhombi are represented in black, crimson, and turquoise, respectively. Right: The “orientation-agnostic” Cr^II^–Cr^III^ percolating network, i.e., where all nearest-neighbor Cr^II^ count toward the percolating network of Cr ions. In contrast, “orientation-restricted” means that only adjacent Cr^II^ ions with orthogonal elongated axes are considered for this network. (**B**) Percolation fraction of interconnected Cr networks within the large-cell LASC simulations as a function of the fractional occupation of Cr^II^ at the *B*-site (*X*_*Cr(II)*_). (**C**) The integrated compositional mixing metric^33^ Σ_*X*_*i*__ as a function of *X*_*Cr(II)*_ for Ag^I^, Cr^III^, and Cr^II^ ions, represented by the solid gray, crimson, and turquoise curves, respectively. Smaller values of Σ_*X*_*i*__ denote better mixing. The error-bars represent the standard deviation of the metric for the ensemble of LASC structures considered at each composition. Here, the dashed black line marks the value of Σ_*X*_*i*__ for random (uncorrelated) *B*-site mixtures, which is ca. 0.8033 for all compositions.^32^ (**D**) Section of a representative image of the LASC simulated alloy **(Ag** ^**I**^ **Cr** ^**III**^ **)­Cr** ^**II**^_**2**_. (**E**) Section of a representative image of a simulated structure derived from (D)—with the *B*-site species shuffled randomly—i.e., the uncorrelated *B*-site mixture. (**F**) Plots of the scaled local mole-fraction variance σ_*X*_*i*__^2^(*R*)/[*X*_*i*_(1 – *X*_*i*_)] as a function of a dimensionless sampling window radius *R*/*a* for the Cr^II^, Cr^III^, and Ag^I^ rhombi in the LASC simulated alloy **(Ag** ^**I**^ **Cr** ^**III**^ **)­Cr** ^**II**^_**2**_, represented by the turquoise, crimson, and gray curves, respectively. The dashed black line is the scaled variance for the random *B*-site mixture. For *R*/*a* > 1, the Cr^II^ sites show worse mixing and the Cr^III^ and Ag^I^ sites show better mixing in the mosaic perovskite, than in an uncorrelated alloy.

Furthermore, via similar analysis of the LASC structures, we estimated that the critical fraction of Cr^II^ needed to support percolating clusters of *only* Cr^II^ at the *B*-sites is in the range 0.92 < *X*_*Cr(II)*_ < 0.95 [**(Ag** ^**I**^ **Cr** ^**III**^ **)­Cr** ^**II**^_***x***_ where 23 < *x* < 38; Figure 4B]. The increase of the percolation thresholds for the all-Cr (*X*_*Cr*_ ca. 0.67) and the Cr^II^-only (*X*_*Cr*_^*II*^ ca. 0.96) networks, over that for a random (Bernoulli) *B*-site network (0.59), can be attributed to the tendency of the Cr^II^ rhombi to aggregate into compact domains as their concentration increases. The large increase of the percolation threshold for the all-Cr network (from 0.59 to 0.67) can also be attributed to the tendency of Cr^III^ and Ag^I^ units to aggregate into domains of endmember double perovskite ordering (Figure 4A and Figure S9, Supporting Information). As a result, **(Ag** ^**I**^ **Cr** ^**III**^ **)**_***y***_ **Cr** ^**II**^_***x***_ alloys where the total mole fraction of Cr is equal to 0.67 but with *y > x* are unlikely to support percolating all-Cr networks due to the tendency of the Cr^III^–Cl units to remain trapped within endmember double perovskite domains.

We then sought to identify the **(Ag** ^**I**^ **Cr** ^**III**^ **)**_***y***_ **Cr** ^**II**^_***x***_ alloy with optimal *B*-site mixing. We probe the relative mixing characteristics of the individual *B*-site ions in the LASC structures here using our recently developed metrics for quantifying the degree of phase mixing and separation in multiphase heterogeneous materials across length scales.^33^ We compute the local *molar-fraction variance*, σ_*X*_*i*__^2^(*R*), of species *i* associated with a circular sampling window of radius *R*. We note that σ_*X*_*i*__^2^(*R*) is a decreasing function of *R* for which the upper bound is σ_*X*_*i*__^2^(0) = *X*_*i*_(1 – *X*_*i*_) and *lim*_*R*__→∞_σ_*X*_*i*__^2^(*R*) = 0. To quantify the overall degree of mixing in the entire system across all length scales, we consider the integrated compositional mixing metric for each rhombus species *i*,^31,32^

3 $$\Sigma_{X_{i}}=\frac{1}{X_{i}\left(1-X_{i}\right)}\int_{0}^{\infty }{\sigma_{X_{i}}}^{2}\left(R\right)dR$$

in which the factor [*X*_*i*_(1 – *X*_*i*_)]^−1^ normalizes the metric such that the alloys can be compared across compositions (see Methods for additional details). In short, smaller values of σ_*X*_*i*__^2^(*R*) and Σ_*X*_*i*__ correspond to a greater degree of mixing across a length scale *R* or the entire system, respectively.^33^

Upon qualitative inspection of the simulated structures, it appears that **(Ag** ^**I**^ **Cr** ^**III**^ **)­Cr** ^**II**^_**2**_ is the most well-mixed, homogeneous alloy (Figure 4A and Figure S9, Supporting Information). Indeed, the integrated compositional mixing metric (eq 3) for the Ag^I^, Cr^II^, and Cr^III^ ions as a function of *X*_*Cr(II)*_ (Figure 4C) shows that the alloys with 0.5 < *X*_*Cr(II)*_ < 0.55 are optimally mixed, with Σ_*X*_*Cr*_ _(*II*)__ ≈ 0.88 and Σ_*X*_*Cr*_ _(*III*)__ ≈ Σ_*X*_*Ag*__(*I*)__ ≈ 0.56. As a reference, the value of Σ_*X*_*Cr*_ _(*II*)__ for this range of compositions is slightly higher than that of an uncorrelated *B*-site mixture (i.e., Σ_*X*_*i*__ ≈ 0.80 for 0 < *X*_*i*_ < 1; Figure 4C).^32^ These observations support conclusions regarding the preferred composition of the Cu^I^–Cu^II^ mosaic perovskite: *A*_8_(Cu^I^In^III^)­Cu^II^_2_Cl_16_,^15^ where this composition (*X*_*Cu(II)*_ = 0.5) was isolated from solutions containing various precursor stoichiometries. We hypothesize that the higher degree of mixing observed in the 0.5 < *X*_*Cr(II)*_ < 0.55 alloys, compared to compositions with other values of *X*_*Cr(II)*_, can be explained as follows: regardless of the bulk composition, Ag^I^ and Cr^III^ units tend to aggregate into domains of the endmember double perovskite, whereas Cr^II^ units aggregate into domains of the endmember single perovskite (see Figure 4A and Figure S9, Supporting Information). Thus, at and around *X*_*Cr(II)*_ = 0.5, there is enough of each species to yield similarly shaped and sized domains and support mixing (Figure S11, Supporting Information). Simulations of the **(Ag** ^**I**^ **Cr** ^**III**^ **)­Cr** ^**II**^ and **(Ag** ^**I**^ **Cr** ^**III**^ **)Cr** ^**II**^_**3**_ alloys show larger domains of endmember double and single perovskite, respectively. This phase-segregation behavior is reflected in the mixing metric: relative to the **(Ag** ^**I**^ **Cr** ^**III**^ **)­Cr** ^**II**^_**2**_ alloy, the Cr^II^ units are about 2.4–3 times more phase-segregated as *X*_*Cr*_ _(*II*)_ → 0.1 and *X*_*Cr*_ _(*II*)_ → 0.9 (Figure 4C). We also note that the values of the mixing metrics for the Ag^I^ and Cr^III^ species are similar to one another across all compositions, reflecting the fact that these rhombi are typically constrained to domains of double perovskite ordering. Overall, the reduced mixing in **(Ag** ^**I**^ **Cr** ^**III**^ **)­Cr** ^**II**^ and **(Ag** ^**I**^ **Cr** ^**III**^ **)­Cr** ^**II**^_**3**_, as well as the optimized mixing at the **(Ag** ^**I**^ **Cr** ^**III**^ **)­Cr** ^**II**^_**2**_ composition, is supported by quantitative analysis of Σ_*X*_*i*__ from the simulated structures (Figure 4C).

Finally, we examine the scaled local mole-fraction variance, σ_*X*_*i*__^2^(*R*)/[*X*_*i*_ (1 – *X*_*i*_)], of each species to quantify mixing in the simulated **(Ag** ^**I**^ **Cr** ^**III**^ **)­Cr** ^**II**^_***x***_ alloys *at specific length scales* (Figures 4D-F and Figure S12, Supporting Information). By quantifying the scaled variance for individual ions in the simulated **(Ag** ^**I**^ **Cr** ^**III**^ **)­Cr** ^**II**^_**2**_ structure and for a hypothetical uncorrelated *B*-site mixture, we find that the variances for Cr^III^ and Ag^I^ are both less (i.e., better mixed) than that of Cr^II^ for all length scales greater than the lattice constant *a* (Figure 4F). These quantitative observations are consistent with qualitative ones: the matrix of Cr^II^ in the LASC structure (Figure 4D) and in an uncorrelated *B*-site mixture (Figure 4E) appear to be equally well mixed. By contrast, Cr^III^ and Ag^I^ are much more likely to cluster among like-species (Cr^III^–Cr^III^ and Ag^I^–Ag^I^) in the random mixture rather than in the LASC structure. Notably, lim_*R*→∞_ σ_*X*_*i*__^2^(*R*) ∼ *R* ^–2^ for the random *B*-site mixture^32^ and all three *B*-site ions in the LASC structures (Figure 4F, inset). Such large-*R* asymptotic decay of density fluctuations is commonly observed in disordered 2D systems.^32,44^

### Magnetic Ground State of the (Ag^I^Cr^III^)­Cr^II^_2_ Alloy

Given the extensive validation of miscibility and Cr percolation provided by the square-lattice LASC simulations of **(Ag** ^**I**^ **Cr** ^**III**^ **)Cr** ^**II**^_**2**_ (Figure 4), we sought to investigate the bulk magnetic properties of this mosaic perovskite. The magnetism of the parent single and double perovskite precursors (see above, Figure 1) provide context for the magnetic properties of the alloy. Using SQUID magnetometry, we probed polycrystalline powders of (BA)_2_CrCl_4_, (BA)_4_AgCrCl_8_, and the **(Ag** ^**I**^ **Cr** ^**III**^ **)Cr** ^**II**^_**2**_ alloy—notably identical to those characterized by UV–vis spectroscopy and X-ray and neutron scattering (Figure 2). Similar to other Cr^II^ perovskites,^9,10,13,14^ ^,^ ^36,37,41,45^ (BA)_2_CrCl_4_ is a soft ferromagnet; isothermal magnetization measurements show open hysteresis with modest coercive fields at temperatures below *T*_*C*_ (Figure S13, Supporting Information). The double perovskite (BA)_4_AgCrCl_8_ is a simple paramagnet, due to the isolation of Cr^III^ centers, with a near-constant susceptibility-temperature product (*χT*) close to the spin-only Cr^III^ effective moment (*μ*_*eff*_; expected, 3.87*μ*_*B*_; measured, 3.85*μ*_*B*_; Figure 5A). In contrast, stronger coupling between Cr centers in the mosaic perovskite **(Ag** ^**I**^ **Cr** ^**III**^ **)­Cr** ^**II**^_**2**_ is evident in variable-temperature DC susceptibility measurements.

**Figure 5:**
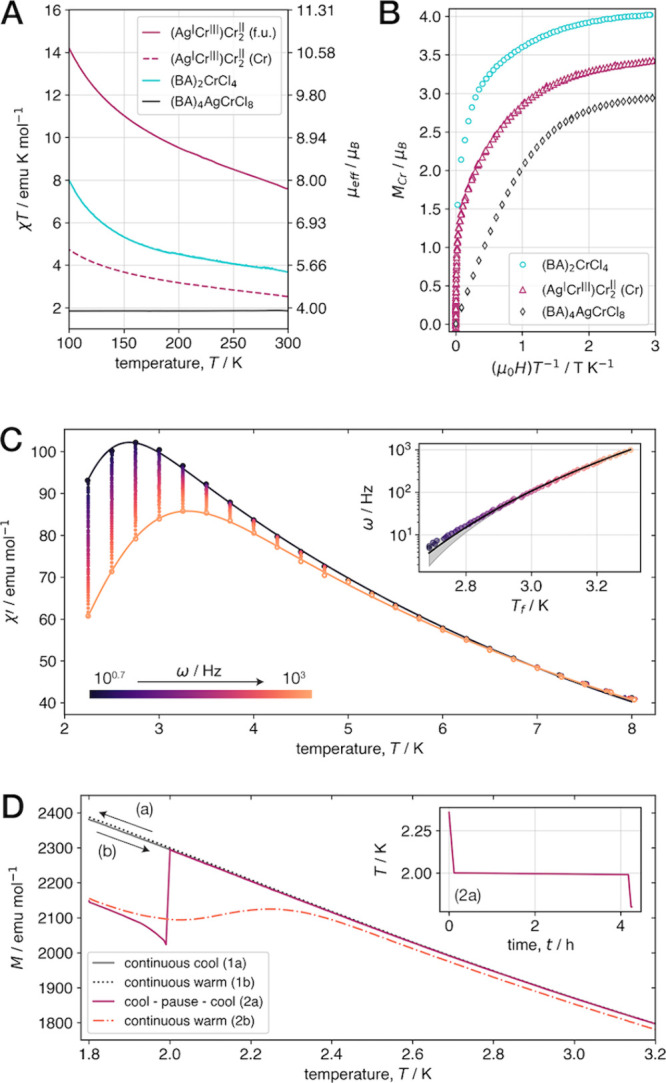
**Bulk magnetic properties and spin-glass dynamics in the (Ag** ^**I**^ **Cr** ^**III**^ **)­Cr** ^**II**^_**2**_ **perovskite**. (**A**) Susceptibility-temperature product (*χT*) determined from variable-temperature measurements with an applied DC field (*μ*_*0*_ *H*) of 1000 Oe [(BA)_4_AgCrCl_8_ and **(Ag** ^**I**^ **Cr** ^**III**^ **)Cr** ^**II**^_**2**_] or 100 Oe [(BA)_2_CrCl_4_]; the effective moment for a paramagnet (*μ*_*eff*_) is related to the susceptibility through $\mu_{eff}=\sqrt{8\chi T}$ ; *χT* for **(Ag** ^**I**^ **Cr** ^**III**^ **)­Cr** ^**II**^_**2**_ was normalized to the formula unit (f.u.) and to the number of Cr ions per f.u. (Cr) for comparison with those of the precursors. (**B**) Isothermal DC variable-field magnetization as a function of reduced field [(*μ*_*0*_ *H*)*T* ^–1^] showing close to the expected saturation magnetization for 4, 3.66, and 3 unpaired electrons per Cr in (BA)_2_CrCl_4_, **(Ag** ^**I**^ **Cr** ^**III**^ **)­Cr** ^**II**^_**2**_, and (BA)_4_AgCrCl_8_, respectively. (**C**) In-phase component of the zero-field AC magnetic susceptibility of **(Ag** ^**I**^ **Cr** ^**III**^ **)­Cr** ^**II**^_**2**_, measured with a drive field of 1 Oe and frequency in the range 5 Hz < ω < 990 Hz; exponential Gaussian fits were used to determine the maximum at *T*_*f*_ (shown for low- and high-ω temperature scans); inset: *T*_*f*_ values (points) and fit to the dynamical scaling relation ω = ω_0_[*T*_*f*_/*T*_*g*_ – 1]^*zv*^ (black line) with a 4σ-uncertainty window (gray). (**D**) Field-cooled DC magnetization measurements of **(Ag** ^**I**^ **Cr** ^**III**^ **)­Cr** ^**II**^_**2**_ (*μ*_*0*_ *H* = 25 Oe) under continuous (1) and cool-pause-cool (2) protocols near *T*_*f*_ (*T*_*g*_); inset: temperature profile during the pause step in (2), where *μ*_*0*_ *H* = 0 Oe.

The *χT* data for **(Ag** ^**I**^ **Cr** ^**III**^ **)­Cr** ^**II**^_**2**_ increase upon cooling from room temperature (Figure 5A), as observed for (BA)_2_CrCl_4_ and other ferromagnetic exchange-coupled layered-perovskite^9,10,14,36^ ^,^ ^37,41,45^ and metal–organic^46,47^ Cr magnets. Indeed, in line with these examples, *χT* exceeds the calculated high-spin *μ*_*eff*_ in the range 100 K < *T* < 300 K [**(Ag** ^**I**^ **Cr** ^**III**^ **)­Cr** ^**II**^_**2**_: expected, 4.56*μ*_*B*_; measured, 4.51*μ*_*B*_ at *T* = 300 K and 6.15*μ*_*B*_ at *T* = 100 K. (BA)_2_CrCl_4_: expected, 4.90*μ*_*B*_; measured, 5.44*μ*_*B*_ at *T* = 300 K and 7.98*μ*_*B*_ at *T* = 100 K]. Thus, *χT* in the paramagnetic phase of **(Ag** ^**I**^ **Cr** ^**III**^ **)­Cr** ^**II**^_**2**_ suggests that, upon alloy formation, Cr^II^ remains in the high-spin state and strong magnetic coupling persists despite Cr^II^ dilution [relative to (BA)_2_CrCl_4_]. Notably, no signature of a bulk phase transition in the **(Ag** ^**I**^ **Cr** ^**III**^ **)­Cr** ^**II**^_**2**_ alloy appears upon cooling from *T* = 100 to 5 K. Isothermal measurements of **(Ag** ^**I**^ **Cr** ^**III**^ **)­Cr** ^**II**^_**2**_ varying the applied field (*μ*_*0*_ *H*) show increasing saturation magnetization upon cooling, as well as negligible hysteresis at *μ*_*0*_ *H* = 0 (Figure S14, Supporting Information).

The moments of (BA)_2_CrCl_4_ and (BA)_4_AgCrCl_8_ each saturate near the expected values of 4*μ*_*B*_ (4 unpaired electrons) and 3*μ*_*B*_ (3 unpaired electrons), respectively (Figure 5B). The expected saturation magnetization of **(Ag** ^**I**^ **Cr** ^**III**^ **)­Cr** ^**II**^_**2**_ can be taken as the average magnetization of two Cr^II^ and one Cr^III^ center (or 3.66*μ*_*B*_), and the experimental data show near-saturation at 3.5*μ*_*B*_ (Figure 5B). Deviations between the calculated and observed moments could originate from minor uncertainty in composition from the mechanochemical synthesis (ca. 3%, see Methods). Notably, the approach to saturation is rapid for the **(Ag** ^**I**^ **Cr** ^**III**^ **)­Cr** ^**II**^_**2**_ alloy as the observed magnetization per Cr ion (*M*_*Cr*_) reaches 50% of the saturation value at a reduced field [(*μ*_*0*_ *H*)*T* ^–1^] of ca. 0.16 T K^–1^, nearer to the corresponding value for (BA)_2_CrCl_4_ (ca. 0.06 T K^–1^) than for (BA)_4_AgCrCl_8_ (ca. 0.65 T K^–1^).

We further investigated the nature of the magnetic ground state of **(Ag** ^**I**^ **Cr** ^**III**^ **)­Cr** ^**II**^_**2**_—in the absence of long-range ferri- or ferro-magnetic ordering—using AC magnetic susceptibility measurements in the range 2 K < *T* < 8 K. Intriguingly, the maximum of an in-phase susceptibility (χ′, Figure 5C) feature exhibits a marked dependence on the frequency (ω) of the driving field, a hallmark of spin-glass character.^48,49^ The freezing temperature, *T*_*f*_, was determined by fitting these χ′(*T*) data (fixed ω) and parametrized as a function of log­(ω),^48^ δ = Δ*T*_*f*_/(*T*_*f*_ Δ­(logω)). The average value of δ was 0.09 within the range 5 Hz < ω < 990 Hz, similar to other insulating spin glasses and below typical values observed from superparamagnetic nanomaterials^48,49^ including amorphous alloys (e.g., δ = 0.14 for Fe_88_Zr_8_B_4_). To relate the spin dynamics to these frequency-dependent data, we fit ω­(*T*_*f*_) to a dynamical scaling relation (Figure 5C, inset), ω = ω_0_[*T*_*f*_/*T*_*g*_ – 1]^*zv*^, where ω_0_ is the characteristic frequency, *T*_*g*_ is the glass-transition (critical) temperature, and *zv* is the critical exponent. At long relaxation times (ω→0), *T*_*f*_ and *T*_*g*_ are equivalent and reflect the true phase transition temperature. The fitted *zv* value of 7.4(5) is also indicative of a strongly coupled insulating spin glass,^49,50^ and the fitted *T*_*g*_ of 2.14(6) K provides an estimate of a finite spin-glass phase-transition temperature. We further verified that ball-milling (BA)_2_CrCl_4_ alone does not produce spin-glass or superparamagnetic signatures (Figures S6 and S15, Supporting Information); thus, we propose that the tiling of the disordered alloy is an essential precursor to support the intrinsic spin-glass state (see Discussion).

Finally, the spin-glass ground state was corroborated by testing a memory hypothesis; here, a DC field-cooled measurement (*μ*_*0*_ *H* = 25 Oe) was paused isothermally below *T*_*f*_ and *T*_*g*_, and the applied field was removed for 4 h (Figure 5D). Upon resumption of the cooling with the applied field, the observed moment had decreased, indicating partial demagnetization at *μ*_*0*_ *H* = 0. Upon subsequent warming, an increase in the moment (near the interruption temperature) is observed, and the warming scan closely reproduces the continuous cooling scans. This rejuvenation-memory signature is unique to the nonequilibrium thermodynamics of glasses—particularly contrasting bulk magnets—where the degenerate nature of the ground state leads to metastable states with correlation lengths that depend on the aging conditions. The small barriers between these states relate directly to the dynamics near *T*_*f*_; thus, the moment is expected to reflect the complex potential-energy landscape established by the aging conditions, such as the rejuvenation observed here upon warming (Figure 5D). In summary, the magnetic ground state of the **(Ag** ^**I**^ **Cr** ^**III**^ **)­Cr** ^**II**^_**2**_ alloy is a spin glass with an apparent glass-transition (critical) temperature in the range 2 K < *T* < 3 K.

### Size, Shape, and Connectivity of the Magnetic Domains

The computationally efficient LASC simulations also allow us to explore the spin glass behavior of the mosaic perovskites across a range of compositions. In particular, quantifying the magnetic domains should separate contributions from random-bond-type and cluster-type spin-glass character. Ferromagnetic domains were defined as clusters consisting of nearest-neighbor Cr^II^ units with orthogonal elongated axes as discussed above (see Figure 1A and Supporting Information for details). Thus, the smallest possible ferromagnetic domain that we consider consists of two nearest-neighbor Cr^II^ rhombi satisfying this condition.

We simulated cells of various mosaic **(Ag** ^**I**^ **Cr** ^**III**^ **)­Cr** ^**II**^_***x***_ alloys in the range 0.1 < *X*_Cr(II)_ < 0.9. The topological connectedness properties of ferromagnetic domains were examined using the two-point *clustering* function *C*_2_(*r*).^51^ Whereas the more commonly used two-point *correlation* function *S*_2_(*r*) for the domains gives the probability that the end points of a randomly oriented line segment of length *r* both fall within *any* ferromagnetic domain, *C*_2_(*r*) is the probability that the end points fall within the *same* domain.^51^ Thus, *C*_2_(*r*) is the contribution to *S*_2_(*r*) that describes the connectedness within a single domain, i.e., *S*_2_(*r*) = *C*_2_(*r*) + *D*_2_(*r*),^51^ where *D*_2_(*r*) measures the probability that the end points fall in *different* domains (Figure S16, Supporting Information). Because of its sensitivity to topological features, *C*_2_(*r*) becomes long-ranged as a system approaches its percolation threshold. Indeed, despite the **(Ag** ^**I**^ **Cr** ^**III**^ **)­Cr** ^**II**^_**3**_ alloy being well-below the percolation threshold for the Cr^II^-only ferromagnetic domains (*X*_Cr(II)_ = 0.6 < 0.92; see Figure 4B), *C*_2_(*r*) for this alloy composition is greater than those for **(Ag** ^**I**^ **Cr** ^**III**^ **)­Cr** ^**II**^_***x***_ (*x* = 1, 2) across all length scales (Figure 6A), indicating that the *x* = 3 composition contains larger, discrete ferromagnetic domains.

**Figure 6:**
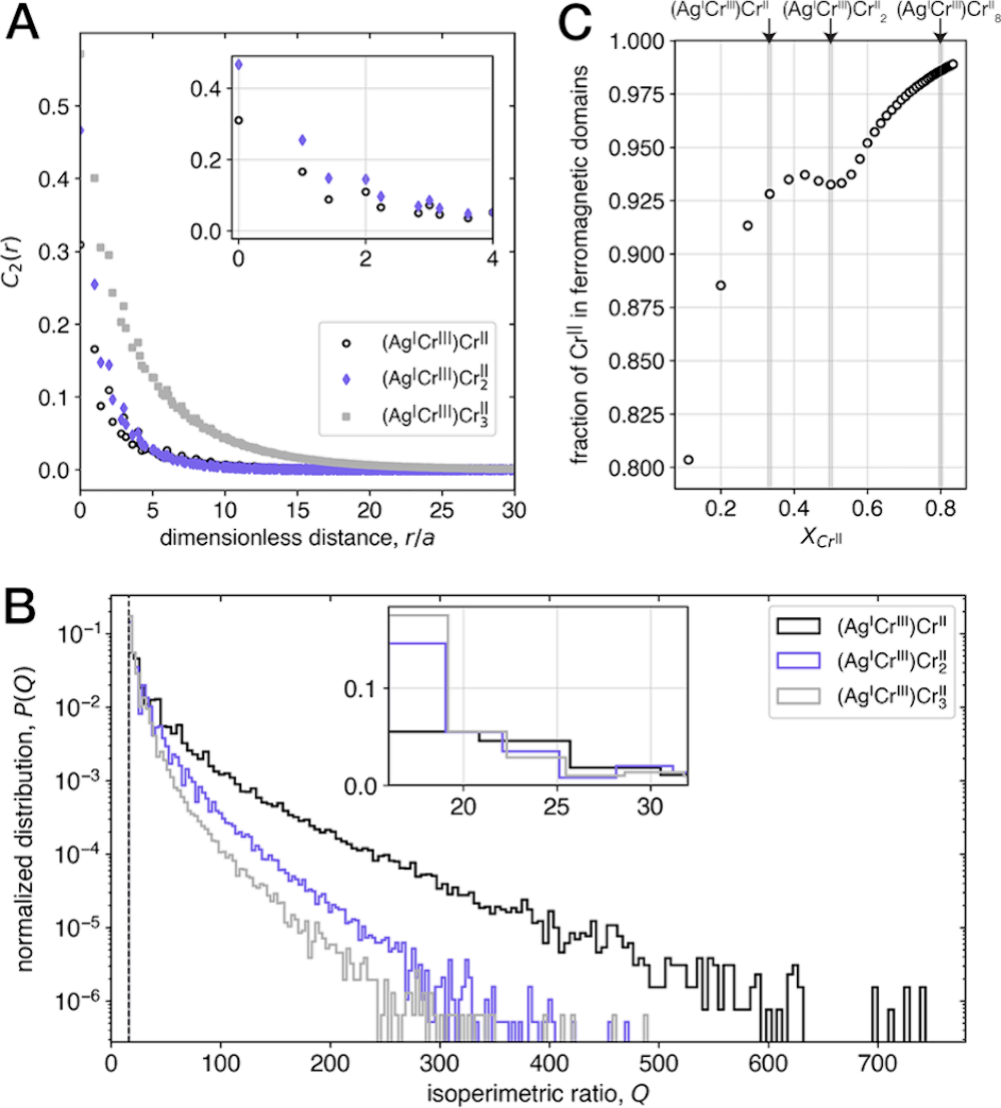
**Composition-dependent miscibility and domain analysis in simulated (Ag** ^**I**^ **Cr** ^**III**^ **)**_***y***_ **Cr** ^**II**^_***x***_ **alloys**. (**A**) Plots of the two-point clustering function, *C*_*2*_(*r*), or the probability that two randomly selected points separated by distance *r* appear in the *same* ferromagnetic domain; inset shows the low-(*r/a*) region of *C*_*2*_(*r*) for the simulated **(Ag** ^**I**^ **Cr** ^**III**^ **)­Cr** ^**II**^_***x***_ (*x* = 1, 2) structures, which suggests that these alloys exhibit similar domain connectedness characteristics. (**B**) Normalized distribution of the isoperimetric ratios, *Q*, for the individual ferromagnetic domains in the three simulated alloys. The dashed vertical line marks the value of the isoperimetric ratio for square domains (*Q* = 16); inset shows the low-*Q* region of the histogram. (**C**) Fraction of Cr^II^ ions (rhombi) within a large-cell LASC simulation that appears in an extended ferromagnetic domain, defined by orthogonal ordering of nearest-neighbor elongated axes (see, e.g., Figure 1A).

The geometrical characteristics of the individual domains were characterized using the isoperimetric ratio, *Q*, which is defined as the ratio between the squared perimeter (*P*) and area (*A*) of the domain, i.e., *Q = P* ^*2*^ */A*. Thus, square domains have *Q* = 16 and elongated rod-like domains have *Q >* 16. We find that the distributions of *Q* for the alloys **(Ag** ^**I**^ **Cr** ^**III**^ **)­Cr** ^**II**^_***x***_ become narrower and shift toward smaller values as *x* increases, reflecting the fact that the ferromagnetic domains become increasingly compact (square) and less ramified (elongated) as *X*_*Cr(II)*_ increases (Figure 6B and Figure S17, Supporting Information). Indeed, the experimentally investigated **(Ag** ^**I**^ **Cr** ^**III**^ **)­Cr** ^**II**^_**2**_ alloy falls in a plateau regime wherein the fraction of Cr^II^ in ferromagnetic domains is between 0.925 and 0.94 (0.33 < *X*_*Cr(II)*_ < 0.55; Figure 6C). This plateau—along with the similar *Q* distributions for the **(Ag** ^**I**^ **Cr** ^**III**^ **)­Cr** ^**II**^ and **(Ag** ^**I**^ **Cr** ^**III**^ **)­Cr** ^**II**^_**2**_ alloys (Figure 6B)—suggests that the growth of ferromagnetic domains is suppressed by a greater tendency for single Cr^II^ units to have Cr^III^ and/or Ag^I^ nearest neighbors for compositions 0.33 < *X*_*Cr(II)*_ < 0.55. Overall, this behavior is consistent with the observation that these compositions are the most well-mixed (Figure 4C). Interestingly, the Cr^II^-rich alloys do not reach 100% Cr^II^ occupation in ferromagnetic domains; taken together with the large (calculated) percolation threshold for Cr^II^ only (Figure 4B), these data suggest that frustrated glasses with huge magnetic moments could be realized at high *X*_*Cr(II)*_.

### Origin of the Spin-Glass Ground State

Among the *B*-site vertex–vertex contacts, there are two ferromagnetic (*J* > 0) exchange pathways (Figure 7A-B): (*i*) ca. 180-degree superexchange between neighboring Cr^II^ centers, wherein a singly occupied *d*_*z* ^2^_ orbital (along the Cr^II^ long axis) points into an unoccupied *d*_*x* ^2^__–*y* ^2^_ orbital (along the Cr^II^ short axis); (*ii*) double exchange in the mixed-valence Cr^II^–Cr^III^ pair, wherein the singly occupied *d*_*z* ^2^_ orbital of Cr^II^ points into an empty *e*_*g*_ orbital of its Cr^III^ neighbor (in the *O*_*h*_ limit, *d*_*z* ^2^_ and *d*_*x* ^2^__–*y* ^2^_ are degenerate and both σ channels contribute indistinguishably). Histograms of the **(Ag** ^**I**^ **Cr** ^**III**^ **)Cr** ^**II**^_**2**_ simulation cells indicate that ca. 71% of the Cr^II^–Cl long bonds and ca. 24% of the Cr^III^ bonds should exhibit ferromagnetic exchange (Figure 7C-D). Exchange coupling between Cr^III^ ions (present at ca. 2%, Figure 7D), and between Cr^II^ ions sharing only long (present at ca. 5%, Figure 7C) or only short bonds, should be antiferromagnetic (*J* < 0). Thus, considerable random-bond disorder^48^ exists to disrupt long-range ferromagnetic ordering. Two classes of canonical random-bond spin glasses, Rb_2_Mn_*x*_Cr_1–*x*_Cl_4_ ^52,53^ and Rb_2_Co_*x*_Cu_1–*x*_F_4_,^54,55^ also crystallize in the layered perovskite structure with in-plane-elongated Cr^II^ and Cu^II^, respectively. However, these spin glasses are homogeneous solid solutions and exhibit only a small frequency shift in the freezing temperature (determined by AC magnetometry).

**Figure 7:**
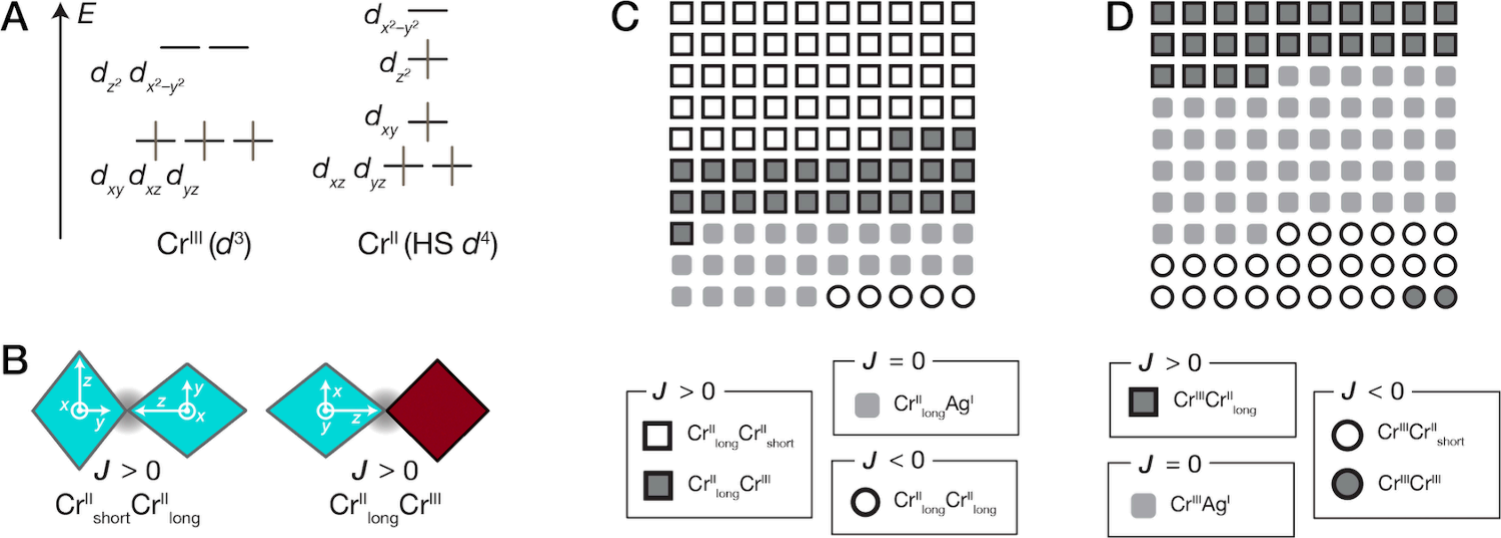
**Statistical analysis of the magnetic exchange pathways**. (**A**) Schematic representation of the *d*-orbital crystal field splitting for six-coordinate Cr ions relevant to this work, octahedral Cr^III^ and tetragonally elongated, high-spin (HS) Cr^II^. (**B**) Ferromagnetic (*J* > 0) exchange pathways in the disordered mosaic alloys, where the local axes of the distorted [Cr^II^Cl_6_] units are overlaid. Histograms of vertex–vertex contacts in LASC simulations of the **(Ag** ^**I**^ **Cr** ^**III**^ **)­Cr** ^**II**^_**2**_ alloy: average populations of the Cr^II^ long-axis (Cr^II^_long_) contacts (**C**) and Cr^III^ contacts (**D**) are shown, along with the expected exchange coupling (ferromagnetic, *J* > 0; antiferromagnetic, *J* < 0; no magnetic coupling, *J* = 0).

In contrast, cluster-type insulating spin glasses such as NaCl-type Eu_*x*_Sr_1–*x*_S^50,56^ exhibit δ values greater than 0.05. For the mosaic **(Ag** ^**I**^ **Cr** ^**III**^ **)**_***y***_ **Cr** ^**II**^_***x***_ alloys [δ = 0.09, **(Ag** ^**I**^ **Cr** ^**III**^ **)­Cr** ^**II**^_**2**_], extensive composition-dependent LASC simulations indicate that ferromagnetic domains exist in all alloys given imperfect mixing (Figure 6C). Ferromagnetic domains in a cluster glass interact weakly through the intervening disordered spins (or diamagnetic ions), resulting in a degenerate ground state where very low temperatures are required to induce cooperative freezing. The spin dynamics inferred from scaling relations relate magnetic correlation lengths in the locally ordered states of **(Ag** ^**I**^ **Cr** ^**III**^ **)­Cr** ^**II**^_**2**_ [*zv* = 7.4(5)] to those of the spin-glass insulator Eu_0.4_Sr_0.6_S^50^ [*zv* = 7–8]. We therefore attribute the ground-state magnetism of **(Ag** ^**I**^ **Cr** ^**III**^ **)­Cr** ^**II**^_**2**_ to a predominant cluster-type spin glass originating from the propensity to form ferromagnetic domains within disordered mosaic alloys.

## Discussion & Outlook

We synthesized a new layered Cr^II^–Cr^III^ chloride perovskite through simple mechanochemical milling of a paramagnetic Ag^I^Cr^III^ double perovskite and ferromagnetic Cr^II^ single perovskite (Figure 1 and Scheme 1), expanding the family of mosaic layered perovskites with three *B*-site ions.

Characterization of this novel mixed-valence alloy suggests that miscibility is mediated by in-plane packing constraints, resulting in numerous local tilting patterns (Figures 2–3). Indeed, the miscibility in the **(Ag** ^**I**^ **Cr** ^**III**^ **)**_***y***_ **Cr** ^**II**^_***x***_ alloys underscores the role of the in-plane Jahn–Teller distortion of high-spin Cr^II^ and augurs further investigation of distorted transition-metal ions in stabilizing mosaic perovskites. We use the LASC simulation algorithm to accurately model the in-plane *B*-site tiling of these complex alloys across length scales, which would be prohibitively expensive to model with *ab initio* calculations, using effective hard-particle packing constraints (Figure 3). The utility and accuracy of LASC modeling of 2D layered mosaic alloys demonstrated here suggest that generalizing the algorithm by adding vertex–vertex interactions to capture the additional packing constraints of 3D mosaic alloys will be a fruitful area of future research.^31^

Magnetic measurements and simulations of the **(Ag** ^**I**^ **Cr** ^**III**^ **)Cr** ^**II**^_**2**_ alloy indicate that the true *B*-site tiling deviates from the idealized ordering that results from orthogonal arrangements of distinct long and short bonds (Figure 3A). Using quantitative mixing and order metrics, we found that the collective arrangement of rhombi in the simulated **(Ag** ^**I**^ **Cr** ^**III**^ **)Cr** ^**II**^_**2**_ alloy exhibits the smallest degree of phase segregation among all compositions considered here (Figure 4). We also observed that in both the Cr^II^-poor and -rich concentration limits, compositional fluctuations in the simulated structures deviate appreciably from those observed in the random *B*-site mixtures (Figure 4), suggesting routes to design analogues with tunable magnetic moments and exchange coupling.

Focusing on the optimally mixed **(Ag** ^**I**^ **Cr** ^**III**^ **)­Cr** ^**II**^_**2**_ alloy, magnetometry measurements revealed a spin glass ground state with a freezing temperature of ca. 3 K (Figure 5). The origin of the spin-glass state (i.e., the competing magnetic exchange interactions that yield near-degenerate ground states) can be explained by the persistence of discrete ferromagnetic domains as well as the combination of antiferromagnetic and ferromagnetic nearest-neighbor couplings (Figure 7). Indeed, beyond explaining the spin-glass ground state, the high-resolution picture provided by our LASC simulations enables quantification of the percolating networks of paramagnetic ions and topological connectedness of the ferromagnetic domains (Figures 4 and 6), suggesting numerous approaches for further tuning of the magnetic ground state by controlling composition-dependent mixing, magnetic exchange pathways, and ferromagnetic domain formation.

An intriguing parallel can be drawn between these **(Ag** ^**I**^ **Cr** ^**III**^ **)**_***y***_ **Cr** ^**II**^_***x***_ alloys and the mixed-valence manganites synthesized at high temperatures^57^ due to the analogous nearest-neighbor ordering and double exchange prevalent in both Mn^III^/ Mn^IV^ and Cr^II^/Cr^III^ pairs. Indeed, regions of the (La_1–*x*_Nd_*x*_)_0.7_Ca_0.3_MnO_3_ phase diagram exhibit extensive short-range order with spin-glass character originating from ferromagnetic domains and a heterogeneous landscape of localized- and itinerant-electron regions in the electronic structure. Further, the **(Ag** ^**I**^ **Cr** ^**III**^ **)**_***y***_ **Cr** ^**II**^_***x***_ mosaic chloride perovskites can be contrasted with the layered A-type antiferromagnet CrCl_3_,^58,59^ which is also synthesized at high temperatures. The edge-sharing [Cr^III^Cl_6_] network in this van der Waals 2D crystal yields ca. 90° ferromagnetic superexchange interactions through the bridging halides;^12,59^ however, interlayer antiferromagnetic coupling outcompetes this intralayer ferromagnetic coupling, requiring isolation of monolayer CrCl_3_ to realize intrinsic 2D ferromagnetism.^60,61^ A distinct advantage of the *A*_2_ *BX*_4_-type perovskites is significant weakening of the interlayer coupling by modulating the interlayer spacing, e.g., from ca. 3.5 Å in CrCl_3_ to 11.1 Å in (BA)_2_CrCl_4_. Thus, while both examples exhibit intralayer ferromagnetic exchange interactions, only the perovskites boast bulk ferromagnetic ordering and synthetic tunability of the interlayer distance. The hybrid organic–inorganic analogues [including (BA)_2_CrCl_4_] obviate the need for exfoliation and offer increased stability^37^ and scalability as bulk materials. Here, the **(Ag** ^**I**^ **Cr** ^**III**^ **)**_***y***_ **Cr** ^**II**^_***x***_ alloys retain the large moment and strong coupling within a percolating perovskite network of paramagnetic Cr ions.

In conclusion, these new mosaic perovskites—and the geometrical and topological analysis provided by the LASC simulations—identify routes toward the inverse design of complex layered halide perovskite magnets. Here, we show how cluster-type spin glasses can be formed through mechanochemical milling at room temperature, with tunable magnetic domains and exchange interactions. We anticipate that similar experimental and computational methods will enable the deliberate synthesis of other complex halide perovskite compositions, with control over local tiling patterns and orbital overlap pathways, to realize emergent properties in a predictable manner.

## Methods

### General Methods

All reagent-grade or higher-purity chemicals were purchased from commercial vendors and used as received unless otherwise noted.

### Synthesis of *n*-Butylamine Hydrochloride, (BA)­Cl

Following an established methodology,^34^ *n*-butylamine (3.3 g, 0.045 mol) was added to 10 mL of ethanol and stirred at 0 °C. Conc. HCl (37% in water; 3.8 mL, 0.045 mol) was added dropwise to the *n*-butylamine solution and subsequently transferred to a crystallization dish. Excess solvent was removed at 85 °C for 12 h, and the resulting product was isolated and dried under reduced pressure.

### Solution-State Crystallization of (BA)_2_CrCl_4_

In a N_2_-filled glovebox, anhydrous chromium­(II) chloride (0.390 g, 3.17 mmol) and (BA)Cl (0.695 g, 6.34 mmol) were combined in 3 mL of conc. HCl (37% in water). The mixture was heated to 100 °C with stirring until the precursors fully dissolved. Colorless plate crystals of the product formed upon cooling to room temperature; the solid was isolated by filtration, washed with an excess of acetonitrile, and dried under reduced pressure. We note that a color change and redissolution of the product (presumably owing to the oxidation of Cr^II^ to Cr^III^ in HCl) was observed after more than 2 h in the mother liquor; rapidly cooling and isolating the product ensured reasonable yield. We also found that powders of the target compound could be synthesized by adapting an established methodology,^11^ where a solution of (BA)Cl (0.612 g, 5.58 mmol; in 4 mL ethanol) was slowly added to a solution of anhydrous chromium­(II) chloride (0.340 g, 2.77 mmol; in 4 mL ethanol) with stirring at room temperature. The solid product was isolated by filtration, washed with an excess of acetonitrile, and dried under reduced pressure. Single crystals of the target compound were grown by a slow vapor diffusion method. Stoichiometric (BA)­Cl and chromium­(II) chloride were dissolved to produce a saturated solution in *N,N’*-dimethylformamide at room temperature; the saturated solution was transferred to an inner shell vial and sealed within a larger 20 mL scintillation vial with an excess of the antisolvent, toluene. Crystals were stored in a N_2_-filled glovebox prior to measurement and transferred to Paratone-N oil for mounting and measuring single-crystal or powder X-ray diffraction. Anal. Calculated for (C_4_H_12_N)_2_CrCl_4_ (%): C, 28.09; H, 7.07; N, 8.19. Found (%): C, 28.36; H, 7.20; N, 8.05.

### Solid-State Synthesis of (BA)_4_AgCrCl_8_

Anhydrous chromium­(III) chloride (0.119 g, 0.750 mmol), silver­(I) chloride (0.108 g, 0.750 mmol), and (BA)Cl (0.329 g, 3.00 mmol) were ground with a mortar and pestle in ambient atmosphere for 5 min before transferring the mixture to a borosilicate glass tube (7 mm OD, 1 mm wall thickness; Chemglass). The tube was subsequently evacuated and sealed with a natural-gas/oxygen flame. The sealed ampule was transferred to an oven at 150 °C for at least 16 h. After cooling to room temperature, the ampule was opened in ambient atmosphere, and the crude product was ground, sealed in an ampule, and heated to 150 °C in repetitive fashion. We note that (*i*) complete conversion to the product was observed after two grind-ampule-heat cycles; (*ii*) trace AgCl was observed in the product when commercial AgCl was used as a precursor, motivating the use of freshly precipitated AgCl—from Ag­(NO_3_) and KCl—stored in a dark, dry atmosphere. Anal. Calculated for (C_4_H_12_N)_4_AgCrCl_8_ (%): C, 25.97; H, 6.54; N, 7.57. Found (%): C, 26.25; H, 6.40; N, 7.37.

### Mechanochemical Synthesis of the Mosaic Perovskite Alloys

In a N_2_-filled glovebox, stoichiometric amounts of (BA)_2_CrCl_4_ and (BA)_4_AgCrCl_8_ were combined and ground in a mortar and pestle for 5 min before loading into a milling cup with zirconia (or yttria-stabilized zirconia) milling balls in a ratio of ca. 1:50 *wt.* precursor mixture: *wt*. milling balls. For instance, for (BA)_8_AgCr_3_Cl_16_ [**(Ag** ^**I**^ **Cr** ^**III**^ **)­Cr** ^**II**^_**2**_], (BA)_2_CrCl_4_ (0.048 g, 0.14 mmol) and (BA)_4_AgCrCl_8_ (0.052 g, 0.07 mmol) were ground and combined with ca. 5 g milling balls. The 12 mL zirconia milling cup was sealed with a PTFE gasket and electrical tape to reduce ingress of oxygen and moisture during milling. Milling was carried out in ambient atmosphere in a Planetary Micro Mill PULVERISETTE 7 (Fritsch) at 800 rpm for 2 h (with a mill: rest ratio of 9 min: 1 min). The cups were promptly reintroduced to inert atmosphere to isolate the product; a typical yield of ca. 50 *wt*.% was recovered from the milling media due to incomplete transfer of the product. The polycrystalline powder product was stored in a N_2_-filled glovebox prior to measurement. Anal. CHN calculated for (C_4_H_12_N)_8_AgCr_3_Cl_16_ (%): C, 26.99; H, 6.79; N, 7.87. Found (%): C, 27.00; H, 6.99; N, 7.70. Anal. Cr:Ag (mol basis) for (C_4_H_12_N)_8_AgCr_3_Cl_16_: calculated (from precursor masses), 2.98; found, 2.90(1) (Table S4, Supporting Information). The 2 h grinding protocol ensured complete conversion—and reproducible syntheses (Figure S18, Supporting Information)—of **(Ag** ^**I**^ **Cr** ^**III**^ **)­Cr** ^**II**^_**2**_ as indicated by (*i*) the absence of features corresponding to a ferromagnetic phase transition from unreacted (BA)_2_CrCl_4_ in the temperature-dependent magnetic property measurements; (*ii*) the absence of crystalline side-phase formation or unnecessary grain-size reduction from excessive milling, determined by PXRD measurements.

### Elemental Analysis

CHN elemental analyses were carried out by Midwest Microlab (Indiana, USA); air-sensitive samples were stored and manipulated in inert atmosphere. Inductively coupled plasma–optical emission spectroscopy (Agilent 5800 ICP–OES) was used to determine the relative composition (digested ratio) of Ag and Cr. Approximately 2 mg of **(Ag** ^**I**^ **Cr** ^**III**^ **)­Cr** ^**II**^_**2**_ powder was dissolved in 11 mL of concentrated HCl (12 M; trace-metal grade). Measurements were performed on the pure digestion as well as a two- and a 10-fold dilution; the reported ratios are pooled from all dilution levels that were measured using the axial detector.

### Powder X-ray Diffraction

Laboratory powder diffraction patterns were collected on a Bruker D8 Advance diffractometer (Cu K_α_ radiation, K_α,1_:K_α,2_ ≈ 2:1; Bragg–Brentano θ-θ geometry). For measurement in reflection geometry, powders were mounted on a zero-background Si stage and rotated continuously during the measurement. Air-sensitive samples were dispersed in Paratone-N oil before transferring to ambient atmosphere and measuring repeatedly until degradation was observed. Supplemental laboratory measurements were collected at the SLAC-Stanford Battery Center on a Bruker D8 Advance (Mo K_α_ radiation, transmission geometry), with powders sealed in borosilicate glass capillaries (ID = 0.5 mm). High-resolution powder X-ray diffraction measurement of (BA)_4_AgCrCl_8_ at beamline 11-BM of the Advanced Photon Source (APS; Argonne National Laboratory) was collected with 27-keV radiation (λ = 0.4597 Å); neat powder was ground in a mortar and pestle then transferred to a Kapton capillary (ID = 1.5 mm) for measurement in transmission (Debye–Scherrer) geometry (μ × r ∼ 0.5). High-resolution powder X-ray diffraction measurement of (BA)_2_CrCl_4_ at beamline 2–1 of the Stanford Synchrotron Radiation Lightsource (SSRL; SLAC National Accelerator Laboratory) was collected with 17-keV radiation (λ = 0.7314 Å); neat powder was ground in a mortar and pestle under a nitrogen atmosphere and then transferred to a borosilicate glass capillary (ID = 0.5 mm) and sealed with epoxy for measurement in transmission geometry. Structure refinements using Pauley and Rietveld refinement methods were carried out using functions within the GSAS-II package.^62^

### Powder X-ray Total Scattering

X-ray total scattering data were collected at beamline 28-ID-1 of the National Synchrotron Light Source II (NSLS-II) at Brookhaven National Laboratory. Powder samples were prepared in sealed 1 mm diameter borosilicate glass capillaries. Scattering patterns were collected at 74.5 keV incident X-ray energy using a PerkinElmer detector with a 31 mm sample-to-detector distance (calibrated using CeO_2_). An empty capillary was used as the background. The samples and background measurements were collected at three distinct positions. Ten scans were collected at each position, each consisting of 15 2-s exposures; no significant evidence of beam-induced sample damage was detected over the full 900-s exposure. Diffuse background scattering subtraction, transmission normalization, and LaPlace noise-filtering were performed on each of the 30 scans per capillary prior to averaging and azimuthal integration of the total scattering intensity, *I­(Q*). The reduced pair distribution function *G­(r)* of each sample was calculated from *I­(Q)* employing the PDFgetX3 Python library for ad hoc reduction to the reduced structure function *F­(Q)* and subsequent Fourier transformation to *G­(r)*.^63^ The reduced pair distribution function *G­(r)* for each sample was generated from the Fourier transform of *F­(Q)* from *Q* = 0.3–21.9 Å^–1^, within the instrumental maximum of 22 Å^–1^.

### Powder Neutron Diffraction

Powders (*ca*. 0.5 g) were ground with a mortar and pestle, loaded, and sealed in Al cans (with a Cu gasket) in a N_2_-filled glovebox. Diffraction patterns were collected on the POWGEN high-resolution neutron powder diffractometer at Oak Ridge National Laboratory, through the mail-in service, with a center wavelength of 2.665 Å at *T* = 100 K. Autoreduced diffraction patterns were analyzed using beamline-standard instrument parameters.

### Single-Crystal X-ray Diffraction

Air-sensitive crystals of (BA)_2_CrCl_4_ were transferred from the mother liquor to Paratone-N oil in a N_2_-filled glovebox and quickly mounted on a Kapton loop under a microscope in ambient atmosphere and transferred to a Bruker D8 Venture diffractometer equipped with a Photon 100 CMOS detector or to the Bruker D85 diffractometer at the Advanced Light Source beamline 11.3.1 or 12.2.1 at Lawrence Berkeley National Laboratory. Frames were collected using ω and φ scans, and unit-cell parameters were refined against all data. The crystals did not show significant decay during data collection. Frames were integrated and corrected for Lorentz and polarization effects using SAINT 8.34a and were corrected for absorption effects using SADABS V2014. Space-group assignments were based upon systematic absences, *E*-statistics, agreement factors for equivalent reflections, and successful refinement of the structures. Structures were solved using the intrinsic phasing method implemented in APEX2. Solutions were refined against all data using the SHELXTL-2013 software package and OLEX2.

### Diffuse Reflectance UV–vis and IR Fourier-Transform Spectroscopy

Diffuse reflectance UV–vis (DR-UV–vis) spectroscopy measurements were collected with a Shimadzu UV-2600 spectrometer in an integrating sphere (λ = 220–1400 nm) with a neat BaSO_4_ background. Air-stable powder samples were ground in a mortar and pestle and diluted in BaSO_4_ before pressing to a compact pellet. Air-sensitive powder samples were ground and diluted in BaSO_4_ in a N_2_-filled glovebox and loaded into an air-free holder behind a transparent silica window and sealed with an O-ring before transferring to ambient atmosphere; repeat measurements were inspected to identify and avoid signatures of degradation. Diffuse reflectance IR Fourier-transform spectroscopy (DRIFTS) measurements were collected with a Bruker Vertex 70 spectrometer (λ^–1^ = 600–5000 cm^–1^) equipped with a liquid-N_2_-cooled HgCdTe detector and a Praying Mantis chamber (Harrick) sample environment. Powder samples were ground in a mortar and pestle and diluted in KBr in a N_2_-filled glovebox. The mixture was quickly transferred to the sample stage under ambient atmosphere to form a pellet before sealing and evacuating the environment. The stage was then heated to ca. 120 °C under vacuum (20 sccm N_2_) to remove adventitious water. When the spectrum stabilized, the stage cooled naturally to room temperature before collecting a final inert-atmosphere measurement.

### Attenuated Total Reflection Fourier-Transform IR Spectroscopy

Attenuated total reflection Fourier-transform IR (ATR-FTIR) measurements were collected with a Thermo Nicolet 6700 spectrometer (λ^–1^ = 500–4000 cm^–1^) equipped with a Smart Orbit ATR accessory. Polycrystalline powders were mounted on the crystal in ambient atmosphere. Spectra were measured continuously for ca. 30 minutes to ensure adventitious hydration by ambient water was not affecting interpretation of spectral features.

### Magnetic Property Measurement

Magnetic properties of the polycrystalline powders were characterized using a SQUID Magnetometer (Quantum Design MPMS3). For the air-stable double perovskite (BA)_4_AgCrCl_8_, powders were loaded and weighed in a plastic cap, then transferred to a brass mount in ambient atmosphere to purge-and-seal the sample environment. For all Cr^II^-containing perovskites, including the single perovskite (BA)_2_CrCl_4_ and the mosaic perovskite alloys, powders were loaded into a quartz tube with eicosane (C_20_H_42_; ca. 1:1 to 1:2 mass ratio of perovskite:eicosane) under Ar atmosphere; loaded tubes were sealed under reduced pressure with a H_2_/O_2_ torch while the sample was submerged in liquid N_2_. Finally, the sealed quartz tube was briefly heated to ca. 45 °C and cooled to dissolve and solidify the eicosane and immobilize the perovskite powder. Diamagnetic contributions from sample holders were subtracted using preset values in the MPMS software; contributions from eicosane and the ions and organic molecules comprising the hybrid perovskites were subtracted using tabulated values.^64^ Formula units for corrections and molar-mass or effective-moment calculations were: (C_4_H_12_N)_2_CrCl_4_, (C_4_H_12_N)_4_AgCrCl_8_, (C_4_H_12_N)_8_AgCr_3_Cl_16_ [**(Ag** ^**I**^ **Cr** ^**III**^ **)­Cr** ^**II**^_**2**_].

### Lattice Adaptive Shrinking Cell (LASC) Simulations

Rhombus dimensions used in the simulations were 5.8 × 5.8 Å, 4.8 × 4.8 Å, and 5.68 × 4.8 Å for Ag^I^, Cr^III^, and Cr^II^, respectively, obtained from single-crystal and powder X-ray diffraction structures. All LASC simulated structures consisted of 200^2^ rhombi occupying a 200 × 200 square lattice. For each stoichiometry, 250 different configurations were produced for ensemble averaging.

### Percolation Threshold, Two-Point Clustering Function, Isoperimetric Ratio, and Mixing Metric Calculations

We numerically estimated the percolation thresholds of the LASC structures by using a burning algorithm^24^ to detect simulation-cell-spanning percolating clusters in the 250 different configurations for each composition. The individual ferromagnetic domains of Cr^II^ in the LASC structures were also identified using a burning algorithm. The two-point clustering functions were then computed from these data using a previously described method^65^ and ensemble averaged over the 250 different configurations for each composition. To compute the isoperimetric ratios, the number of Cr^II^ units within each Cr^II^ domain was taken as a domain’s area. The domain perimeters were computed by counting the number of nearest neighbors of each Cr^II^ unit that were *not* within the domain (e.g., a Cr^II^ with three nearest-neighbors inside the domain and one nearest-neighbor outside of the domain contributes +1 to the domain perimeter). The three histograms of *Q* values in Figure 6 were computed from 250 LASC configurations each. The integrated compositional mixing metrics Σ_*X*_*i*__ were computed from local variances σ_*X*_*i*__^2^(*R*) that were computed from the LASC structures using a previously described method.^33^ For our finite-sized LASC systems, we took the upper limit of integration in eq 3 to be *L*/8 where *L* = 200 is the side-length of the periodic simulation cell.^66^ Mixing metric results were ensemble averaged over the 250 different LASC configurations for each composition.

## Supplementary Material



## Data Availability

The data that support the findings of this study are openly available in the Stanford Digital Repository at https://purl.stanford.edu/xb532rx3275.

## Notes

Supplementary Methods, Supplementary Figures (S1–S18), Supplementary Tables (S1–S4), Supplementary References (PDF)
