# Supplementary material for: A Mosaic Layered Halide-Perovskite Spin Glass: Mechanochemical Alloying of a Ferromagnet and a Paramagnet
Source: ACS Cent Sci. 2026 Jun 24;12(7):981–95. doi: 10.1021/acscentsci.6c00194 (PMC13397285; doi:10.1021/acscentsci.6c00194)
Supplement: Supplementary file 1 [file oc6c00194_si_001.pdf]

Supporting Information

A mosaic layered halide-perovskite spin glass: Mechanochemical alloying of a ferromagnet and a paramagnet

Julian A. Vigil,[a, b, c ‡] Murray Skolnick,[d ‡] Clara Zwanziger,[a] Jiayi Li,[a] Damara Dayton,[e] Michael F. Toney,[e, f, g] Salvatore Torquato[d, h, i, j] * and Hemamala I. Karunadasa [a, k] **

[a] Department of Chemistry, Stanford University, Stanford, California 94305, United States
[b] Department of Chemical Engineering, Stanford University, Stanford, California 94305, United States
[c] College of Chemistry, University of California, Berkeley, Berkeley, California 94720, United States
[d] Department of Chemistry, Princeton University, Princeton, New Jersey 08544, United States
[e] Department of Chemical and Biological Engineering, University of Colorado Boulder, Boulder, Colorado 80309, United States
[f] Materials Science and Engineering, University of Colorado, Boulder, Boulder, CO 80303, United States
[g] Renewable and Sustainable Energy Institute (RASEI), University of Colorado Boulder, Boulder, Colorado 80309, United States
[h] Princeton Materials Institute, Princeton University, Princeton, New Jersey 08544, United States
[i] Department of Physics, Princeton University, Princeton, New Jersey, 08544, United States
[j] Program in Applied and Computational Mathematics, Princeton University, Princeton, New Jersey, 08544, United States
[k] Stanford Institute for Materials and Energy Sciences, SLAC National Accelerator Laboratory, Menlo Park, California 94025, United States
*e-mail: torquato@princeton.edu
**e-mail: hemamala@stanford.edu
‡ these authors contributed equally

Table of Contents:

Supplementary Methods S2

Supplementary Figures S3

Supplementary Tables S22

Supplementary References S24

Supplementary Methods

The Lattice Adaptive Shrinking Cell (LASC) Algorithm. In this section, we provide methodological details concerning the LASC algorithm to accompany our prior report[1] and the details provided in the Methods section (see main text). As the modeling presented in the main text concerns a single inorganic plane within a layered halide-perovskite crystal, the implementation described below assumes that the systems exist in two-dimensional Euclidean space $\mathbb{R}^2$.

The LASC algorithm is a Monte Carlo scheme that generates arrangements of perovskite B-site rhombi with minimal nearest-neighbor vertex-vertex mismatch given a prescribed composition and system size. Note that the rhombi are taken to be effectively rigid, and their geometric centers are fixed to an $L \times L$ square lattice, where $N = L^2$ is the total number of rhombi. Although no out-of-plane octahedral tilting is permitted, Jahn-Teller elongated species (here, Cr^{II}) can make discrete, 90-degree rotations in the plane. The simulation cell is subject to periodic boundary conditions to minimize boundary effects. **Figure S6A** and **Figure S6C** schematically depict two different cases of nearest-neighbor vertex-vertex mismatch. As stated in the main text, we hypothesize that such mismatches, when large enough, are accompanied by instabilities that originate from non-ideal coordination through bridging halide ligands. In contrast, the bonded interaction depicted in **Figure S6B** should be more stable.

Two useful benchmark tests for the LASC algorithm are to recreate the known B-site configurations for the layered Cr^{II} single and $Ag^{I}Cr^{III}$ double perovskite crystals. **Figure S6** depicts images of $N = 10^2$ rhombi arrangements generated via the LASC algorithm for the aforementioned single (**Figure S6D**) and double perovskite (**Figure S6E**) materials. The simulated structures are consistent with the experimental X-ray structure solutions (see Tables S1-S2 and main text, **Figure 1A-B**). Additional LASC simulation details are provided in Ref. 1.

Supplementary Figures

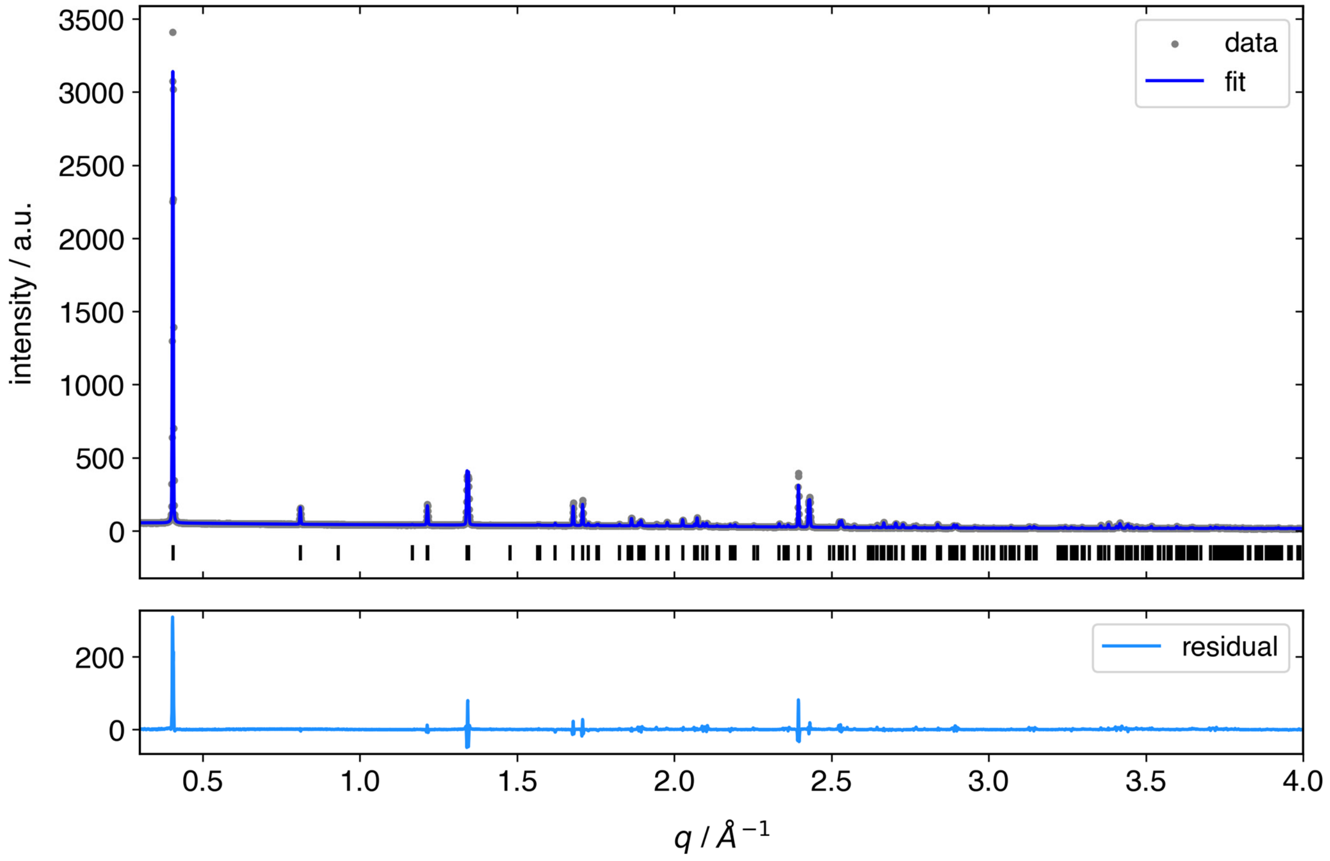

Figure S1. High-resolution synchrotron powder X-ray diffraction pattern of $(BA)_2CrCl_4$ (BA = *n*-butyl ammonium; T = 298 K; λ = 0.7314 Å) and the corresponding best-fit Rietveld refinement over a fitted background (using GSAS-II[2]). Positions of the allowed Bragg reflections are represented with offset black lines. Details of the structure solution are provided in Table S1.

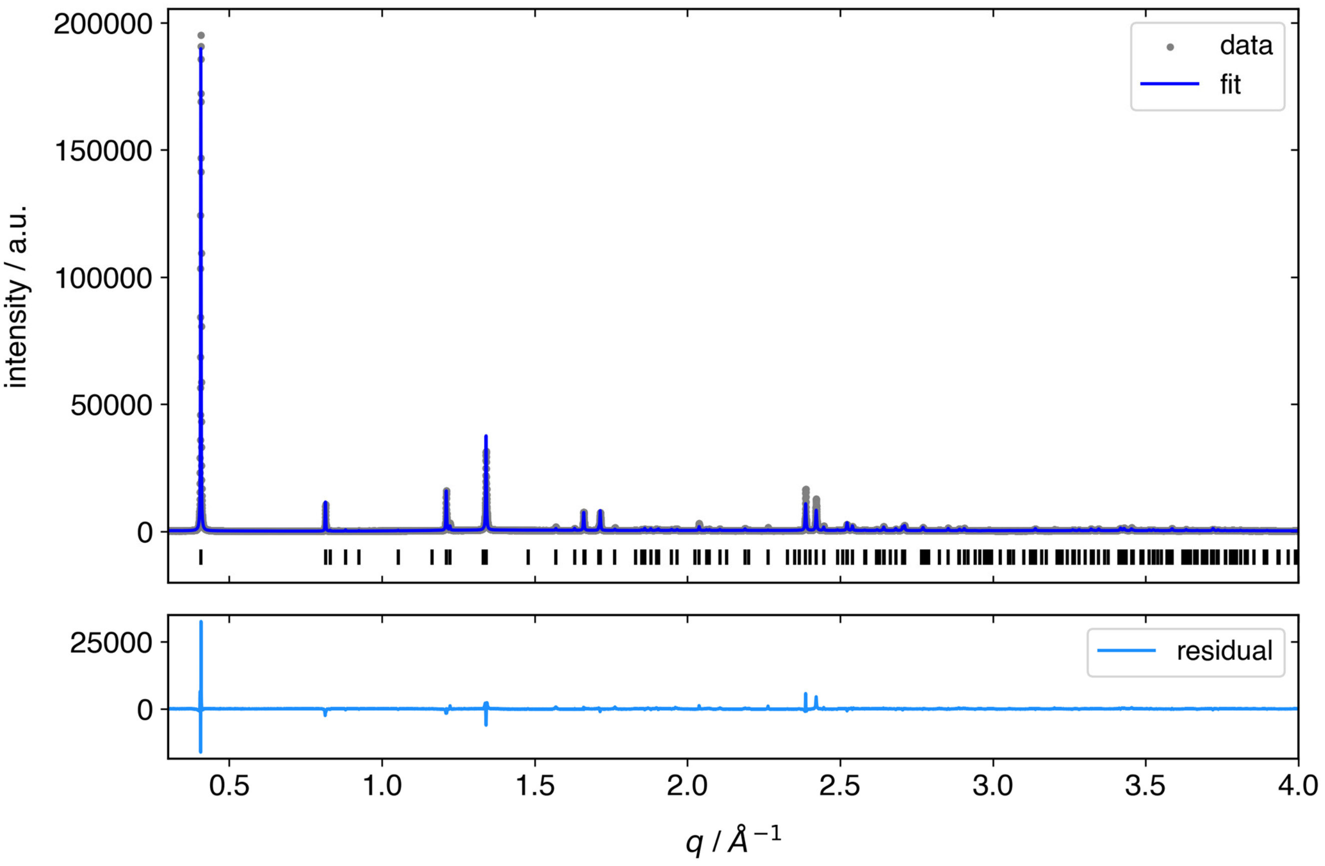

Figure S2. High-resolution synchrotron powder X-ray diffraction pattern of $(BA)_4AgCrCl_8$ (BA = *n*-butyl ammonium; T = 295 K; λ = 0.4597 Å) and the corresponding best-fit Rietveld refinement over a fitted background (using GSAS-II[2]). Positions of the allowed Bragg reflections are represented with offset black lines. Details of the structure solution are provided in Table S2.

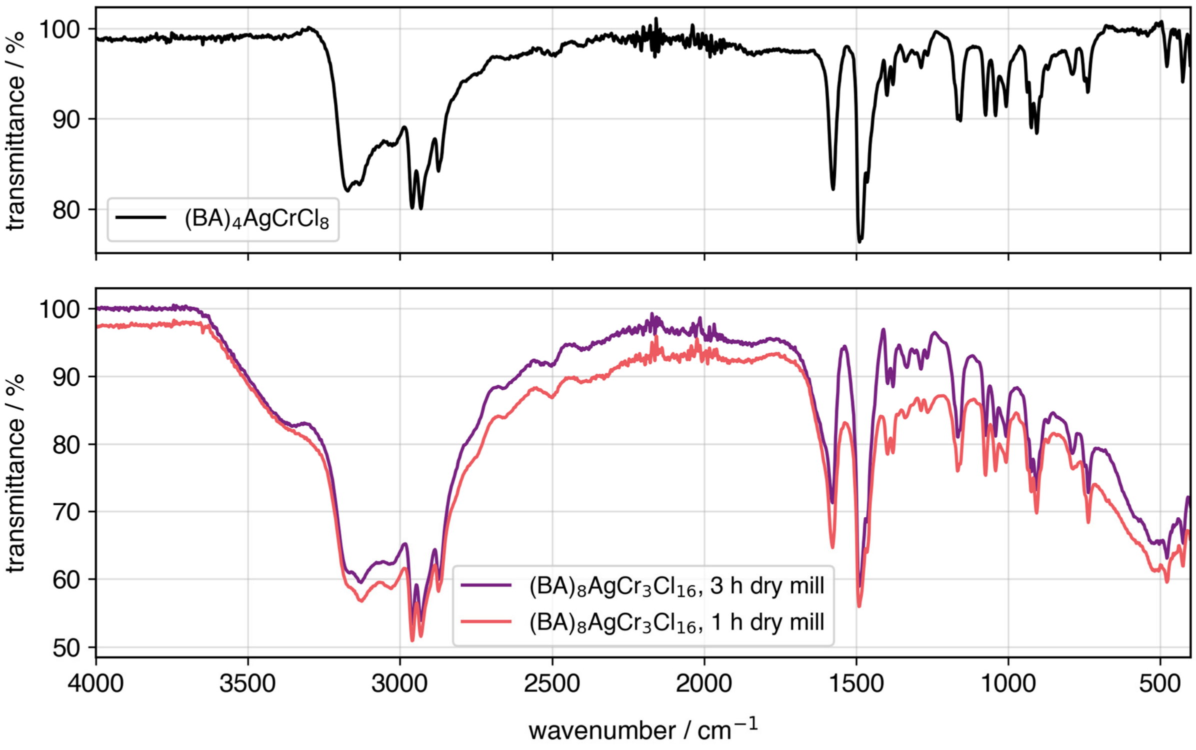

Figure S3. ATR-IR spectroscopy measurements (in ambient atmosphere) of the layered double perovskite [$(BA)_4AgCrCl_8$, top] and the **$(Ag^{I}Cr^{III})Cr^{II}_2$** alloy [$(BA)_8AgCr_3Cl_{16}$, bottom] under various milling conditions (see Methods).

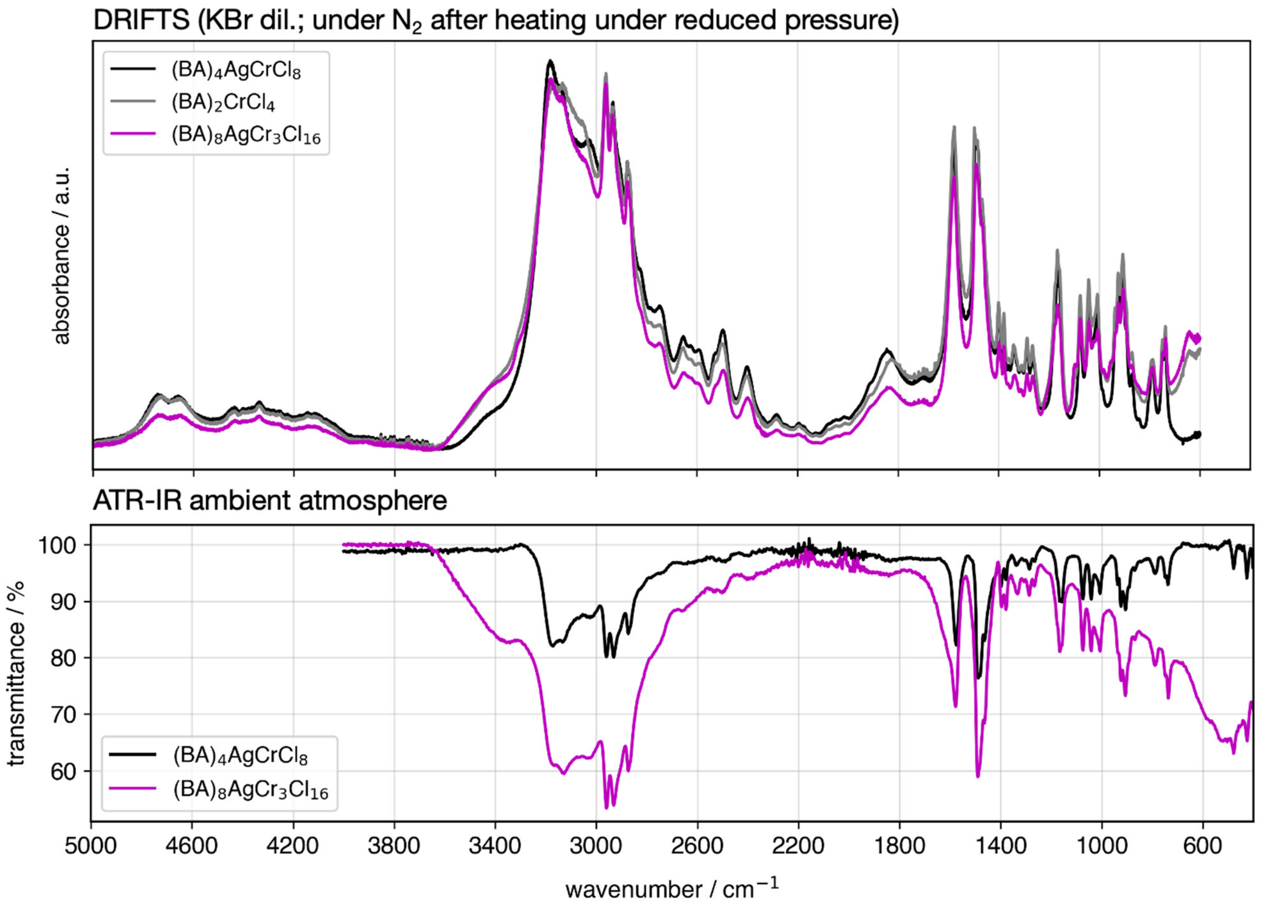

Figure S4. IR spectroscopy measurements of the single perovskite [$(BA)_2CrCl_4$], double perovskite [$(BA)_4AgCrCl_8$], and the **(Ag^ICr^{III})Cr^{II}_2** alloy [$(BA)_8AgCr_3Cl_{16}$] using DRIFTS (top) and ATR-IR (bottom) under the indicated atmosphere and described in detail in the Methods.

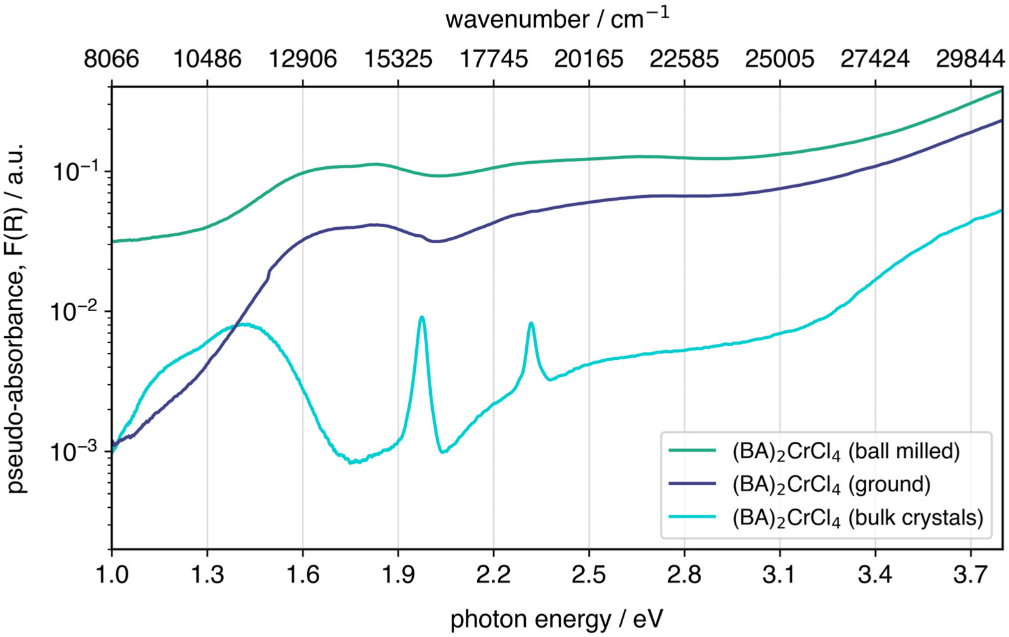

Figure S5. Diffuse reflectance UV-Vis spectra of bulk $(BA)_2CrCl_4$ crystals (data reproduced from Figure 2D, see main text), compared to the corresponding spectra after mortar-and-pestle grinding (under N_2 atmosphere) or after ball milling (following conditions from the synthesis of the mosaic alloys, see Methods); pseudo-absorbance was calculated from the measured reflectance, *R*, using the Kubelka-Munk transformation.[3,4]

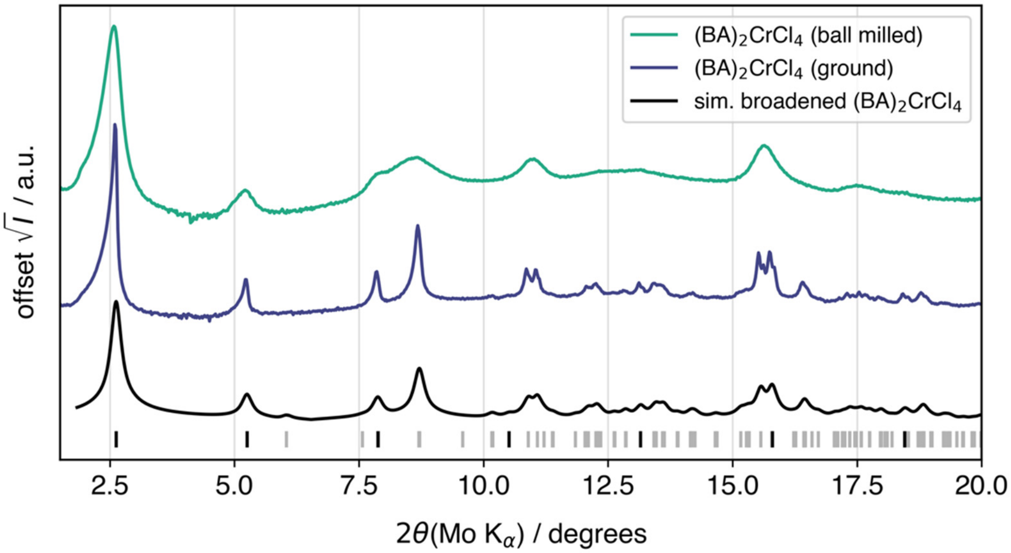

Figure S6. Powder X-ray diffraction patterns from $(BA)_2CrCl_4$ crystals after mortar-and-pestle grinding (under N_2 atmosphere) or after ball milling (following conditions from the synthesis of the mosaic alloys, see Methods); allowed Bragg reflections and a simulated pattern from the structure solution of $(BA)_2CrCl_4$ are shown for comparison [bolded tick marks are associated with the largest interplanar spacing, (*h*00)].

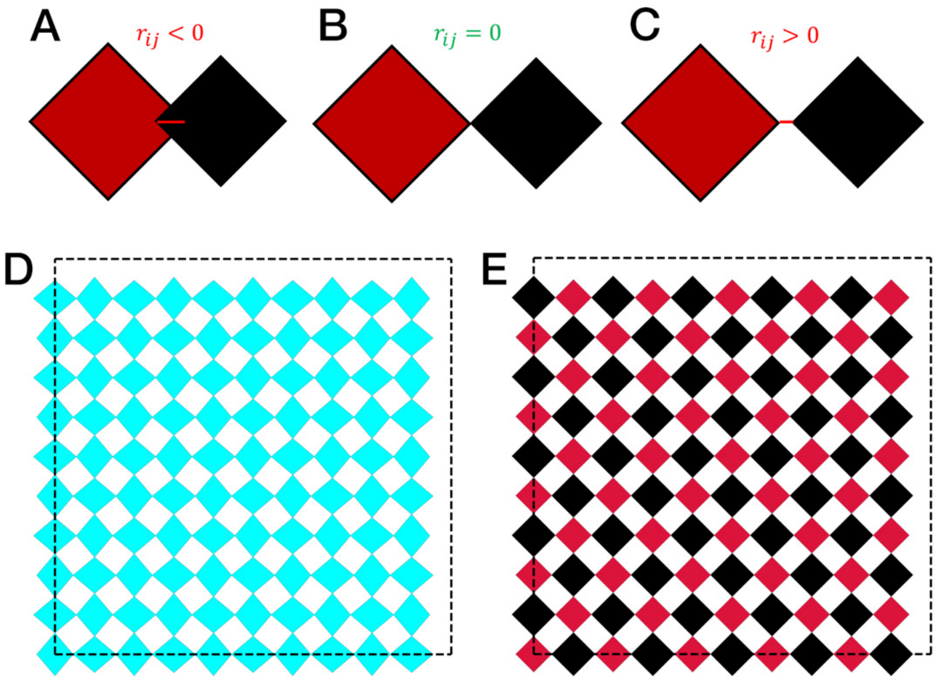

Figure S7. Top: A schematic depicting (**A**) unfavorable compressed, (**B**) favorable bonded, and (**C**) unfavorable stretched *B*-site rhombi nearest-neighbor vertex-vertex interactions. In cases (A) and (C), the nonzero vertex-vertex distance r_{ij} is represented by a red line. Bottom: Images of $N = 10^2$ rhombi arrangements generated by the LASC algorithm for the (**D**) Cr^{II} single and (**E**) $Ag^{I}Cr^{III}$ double perovskites**.** In (**D**) and (**E**), the dashed line denotes the periodic simulation cell boundaries and Cr^{II}–Cl, Ag^{I}–Cl, and Cr^{III}–Cl units are represented by turquoise, black, and crimson rhombi, respectively. These structures predicted by LASC are consistent with the ordering that manifests in X-ray diffraction structure solutions (see main text, **Figure 1A-B**).

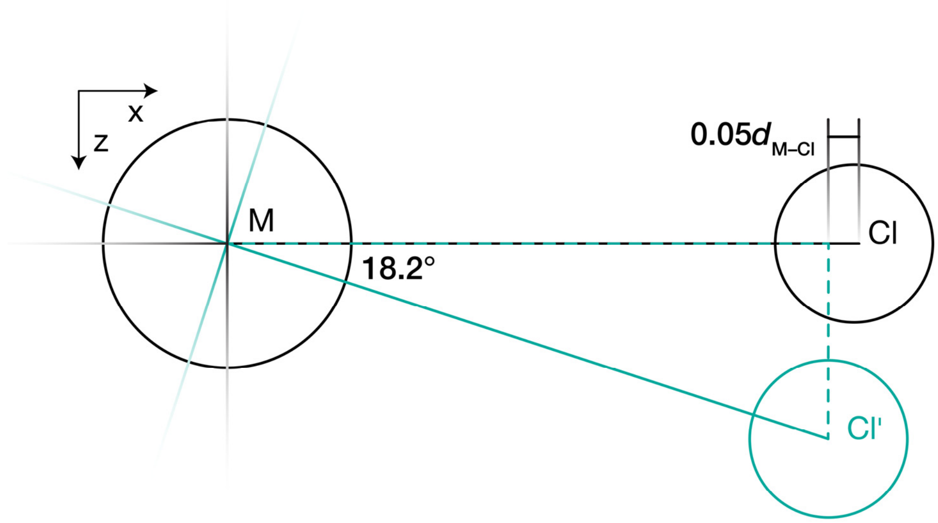

Figure S8. Schematic representation of a fragment of a metal-chloride octahedron before (*M*–Cl, black) and after (*M*–Cl′, teal) a tilt in the *xz* plane that maintains the bond length. A large tilt angle (18.2°) is required to decrease the projection of the M–Cl′ bond along *x* by 5%. This hypothetical tilt represents an out-of-plane distortion (here, the perovskite sheet lies in *xy* plane) often observed in two-dimensional perovskites.

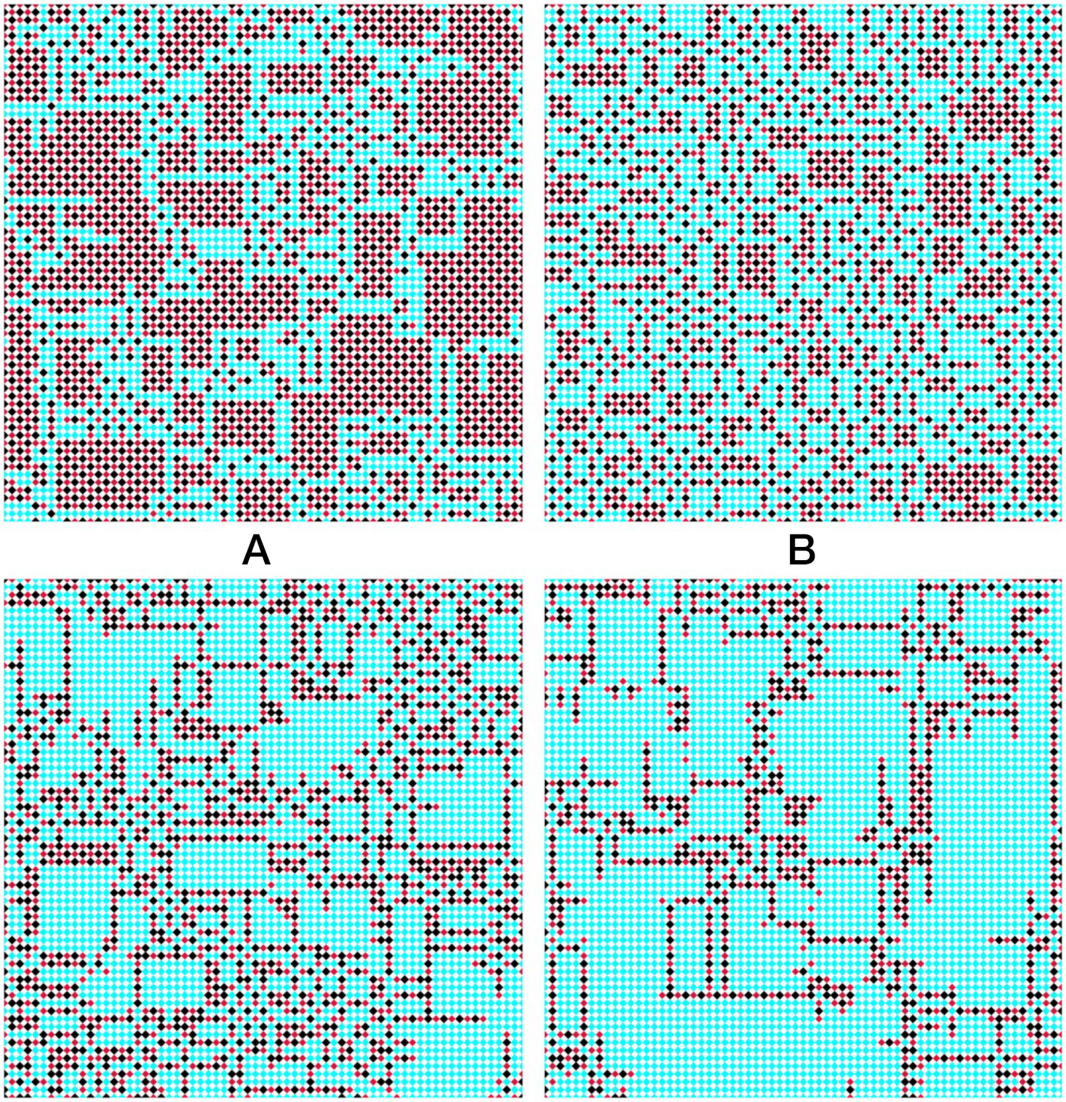

A B C D

Figure S9. Representative portions of $N = 200^2$ rhombi arrangements generated by the LASC algorithm for mosaic alloys $(Ag^{I}Cr^{III})Cr^{II}_{x}$ with compositions (**A**) $x = 1$, (**B**) $x = 2$, (**C**) $x = 3$, and (**D**) $x = 4$. For composition (A), we observe branch-like ferromagnetic domains of $\mathbf{Cr^{II}}$ embedded in a matrix of $\mathbf{Ag^{I}Cr^{III}}$ double perovskite. The most well-mixed composition (B) (see **Figure 4C** and accompanying discussion, main text) consists of uniformly distributed domains of $\mathbf{Cr^{II}}$ single perovskite and $\mathbf{Ag^{I}Cr^{III}}$ double perovskite with similar shapes and sizes. As the concentration of Cr^{II} increases going to systems (C) and (D), we observe that the $\mathbf{Cr^{II}}$ ferromagnetic domains become larger and more compact while remaining separated (i.e., topologically disconnected) by thin (1-2 *B*-site) thick strips of $\mathbf{Ag^{I}Cr^{III}}$ double perovskite. The areas shown in images (A)-(D) represent 1/9th of the total area of the simulation cell. In all images, the Cr^{II}–Cl, Ag^{I}–Cl, and Cr^{III}–Cl units are represented by turquoise, black, and crimson rhombi, respectively.

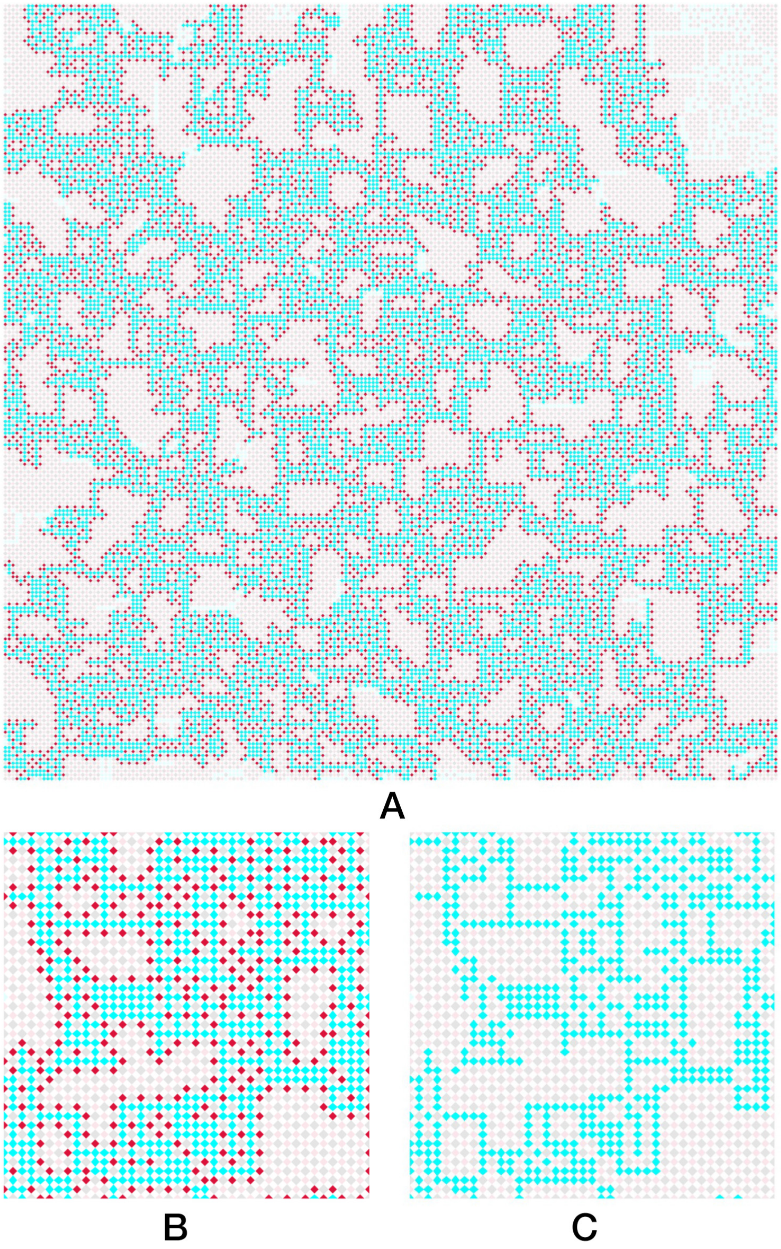

Figure S10. The $N = 200^2$ rhombi configuration of the mosaic alloy $(Ag^{I}Cr^{III})Cr^{II}$ generated by the LASC algorithm with (**A**) the entire orientation-restricted Cr^{II}–Cr^{III} percolating network (see main text for definition, Figure 4 and associated discussion) highlighted, as well as enlarged portions of the image in (A) with (**B**) both Cr^{II} and Cr^{III} and (**C**) only Cr^{II} rhombi highlighted. From images (A) and (B), we see that the orientation-restricted $\mathbf{Cr^{II}}$–$\mathbf{Cr^{III}}$ percolating network consists of thin (2-4 rhombi in width) branched domains of $\mathbf{Cr^{II}}$ units (turquoise) that are surrounded by a single layer of $\mathbf{Cr^{III}}$ units (crimson) from domains of endmember $\mathbf{Ag^{I}Cr^{III}}$ double perovskite. If the Cr^{III} units are removed from the percolating network, as depicted in image (C), we are left with topologically disconnected domains of $\mathbf{Cr^{II}}$. Thus, the percolating network of paramagnetic Cr ions consists of $\mathbf{Cr^{II}}$ domains that are linked together by single $\mathbf{Cr^{III}}$ units. The area shown in (A) here represents the entire 200×200 simulation cell, whereas those shown in (B) and (C) correspond to only 1/25th of the simulation cell.

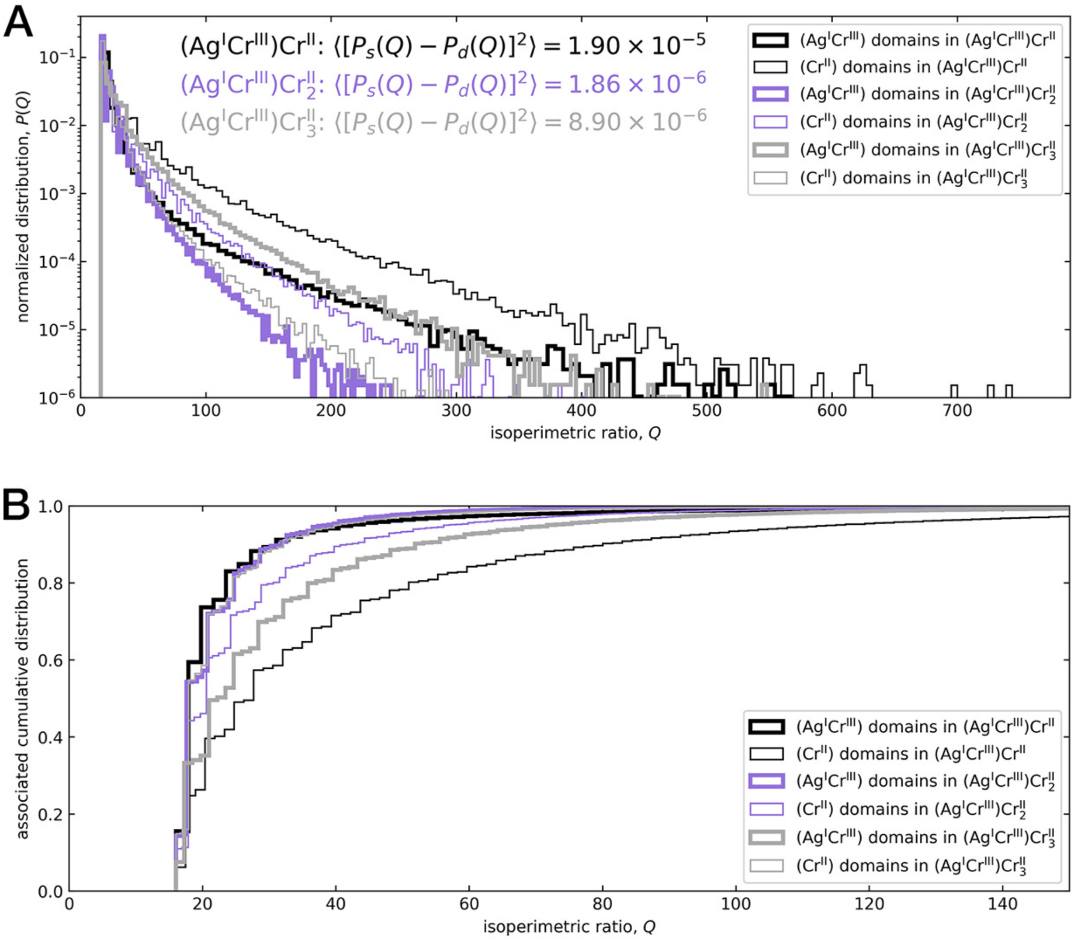

Figure S11. Plots of the (**A**) normalized probability $P(Q)$ and (**B**) associated cumulative distributions of isoperimetric ratios Q (see main text, **Simulating mosaic alloys across various length scales**) for the grains of endmember double perovskite (Ag^ICr^{III}) (thick lines) and single perovskite Cr^{II} (thin lines) in the **(Ag^ICr^{III})Cr^{II}_x** alloys where the x=1, 2, and 3 compositions are highlighted in black, purple, and light gray, respectively. As seen from these plots of $P(Q)$ and its associated cumulative function, the distributions of the double- and single-perovskite domain morphologies (i.e., $P_d(Q)$ and $P_s(Q)$, respectively) are the most similar to one another in the **(Ag^ICr^{III})Cr^{II}_2** alloy. In contrast, the domain morphology distributions are the most dissimilar for the **(Ag^ICr^{III})Cr^{II}** alloy. These differences can be quantified via the mean squared-difference of the distributions, $\langle [P_s(Q) - P_d(Q)]^2 \rangle$ where $\langle \ldots \rangle$ denotes averaging over Q values. We find that $\langle [P_s(Q) - P_d(Q)]^2 \rangle$ is nearly an order of magnitude lower for the **(Ag^ICr^{III})Cr^{II}_2** alloy than it is for the other two compositions. Thus, the LASC simulated **(Ag^ICr^{III})Cr^{II}_2** alloys are the most well-mixed among all compositions considered since the morphologies of their constituent endmember double- and single-perovskite domains are the most similar.

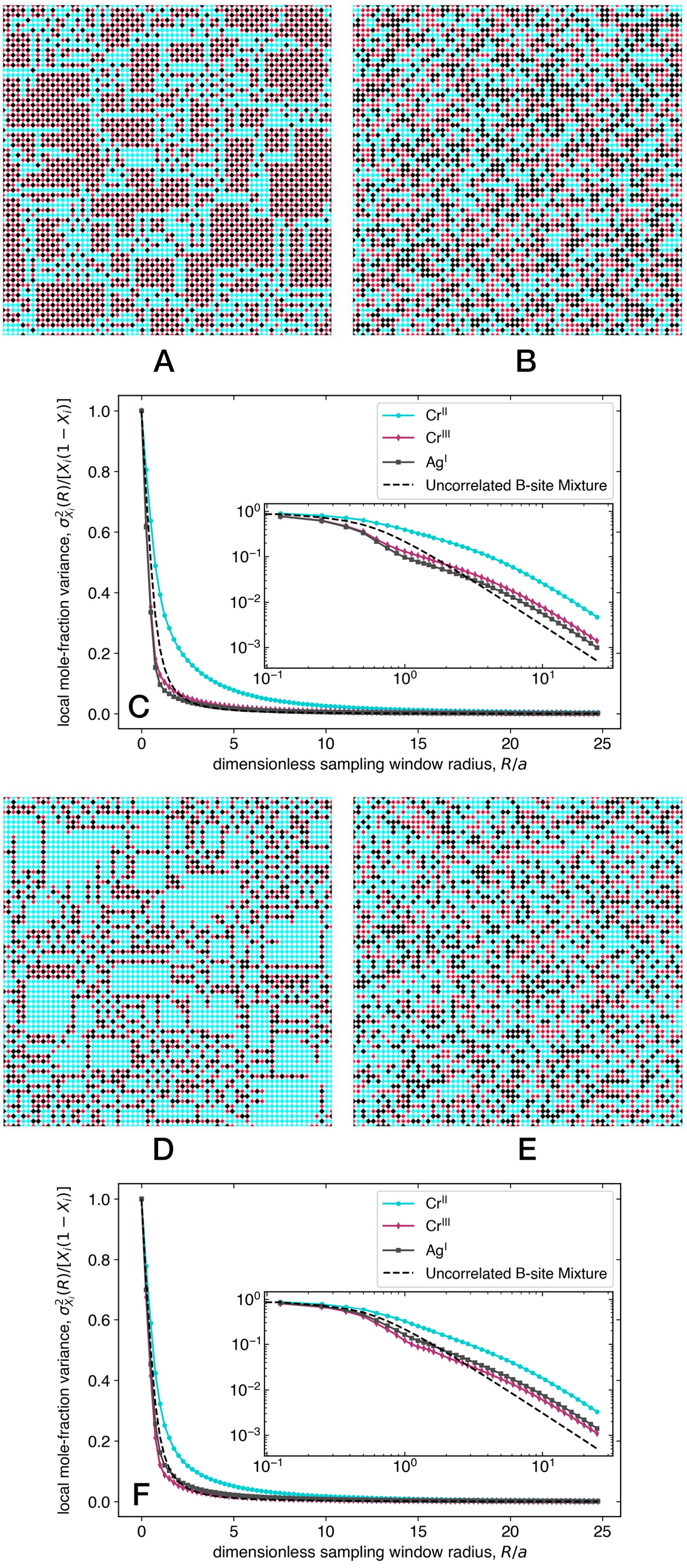
A
B
C
Cr^{II}
Cr^{III}
Ag^{I}
Uncorrelated B-site Mixture
local mole-fraction variance, $\sigma^2_{X_i}(R)/[X_i(1-X_i)]$
dimensionless sampling window radius, R/a
D
E
F
Cr^{II}
Cr^{III}
Ag^{I}
Uncorrelated B-site Mixture
local mole-fraction variance, $\sigma^2_{X_i}(R)/[X_i(1-X_i)]$
dimensionless sampling window radius, R/a

Figure S12. Comparing the degrees of mixing of simulated $(\mathbf{Ag^{I}Cr^{III}})\mathbf{Cr^{II}}$ and $(\mathbf{Ag^{I}Cr^{III}})\mathbf{Cr^{II}_3}$ alloys with corresponding random (uncorrelated) $\boldsymbol{B}$-site mixtures across length scales. Panels (**A**-**C**) correspond to the $(\mathbf{Ag^{I}Cr^{III}})\mathbf{Cr^{II}}$ alloy, and panels (**D**-**F**) correspond to the $(\mathbf{Ag^{I}Cr^{III}})\mathbf{Cr^{II}_3}$ alloy. Images (A) and (D) are the LASC structures, whereas (B) and (E) are images of the corresponding random B-site mixtures. The scaled local mole-fraction variances $\sigma_{X_i}{}^2(R)/[X_i(1-X_i)]$ as a function of the dimensionless sampling window radius R/a are plotted in panels (C) and (F), where a is the distance between nearest-neighbor B-sites. Here, the data for Cr^{II}, Cr^{III}, and Ag^{I} rhombi are represented by the turquoise, crimson, and black curves, respectively. The dashed black line is the scaled local mole-fraction variance for the random B-site mixture. For both alloy compositions, we see in the plots of $\sigma_{\mathrm{Cr(II)}}{}^2(R)$ in panels (C) and (F) that the arrangement of Cr^{II} units is between 2-5 times *more* phase-segregated across all length scales than that found in a random mixture. This behavior is attributed to the tendency of Cr^{II} to cluster among like-species within domains of endmember Cr^{II} single perovskite as seen in the simulated structures (A) and (D), rather than being more uniformly distributed as in the random mixtures shown in (B) and (E). For the $(\mathbf{Ag^{I}Cr^{III}})\mathbf{Cr^{II}}$ alloy, we see in the plots of the metrics $\sigma_{\mathrm{Cr(III)}}{}^2(R)$ and $\sigma_{\mathrm{Ag(I)}}{}^2(R)$ in panel (C) that the arrangements of Ag^{I} and Cr^{III} rhombi are up to two times more mixed than those found in random mixtures for length scales $R < 2a$. This observation reflects the tendency of these rhombi to be found within well-mixed *crystalline* domains of endmember $Ag^{I}Cr^{III}$ double perovskite as seen in the simulated structure (A). As the size and extent of these well-mixed crystalline $Ag^{I}Cr^{III}$ double perovskite domains is reduced in the $(\mathbf{Ag^{I}Cr^{III}})\mathbf{Cr^{II}_3}$ alloy (D), we see that the metrics $\sigma_{\mathrm{Cr(III)}}{}^2(R)$ and $\sigma_{\mathrm{Ag(I)}}{}^2(R)$ plotted in panel (F) are less than that for a random mixture only for length scales $R < a$. For larger length scales that encompass both $Ag^{I}Cr^{III}$ double-perovskite and Cr^{II} single-perovskite domains (i.e., $R \geq ma$, where $m = 1$ for $(\mathbf{Ag^{I}Cr^{III}})\mathbf{Cr^{II}}$ and $m = 2$ for $(\mathbf{Ag^{I}Cr^{III}})\mathbf{Cr^{II}_3}$), the arrangement of Ag^{I} and Cr^{III} rhombi becomes about 10 % more phase-segregated than those in a random mixture. The areas shown in images (A), (B), (D), and (E) represent 1/9th of the total area of the simulation cell. In all images, the Cr^{II}–Cl, Ag^{I}–Cl, and Cr^{III}–Cl units are represented by turquoise, black, and crimson rhombi, respectively.

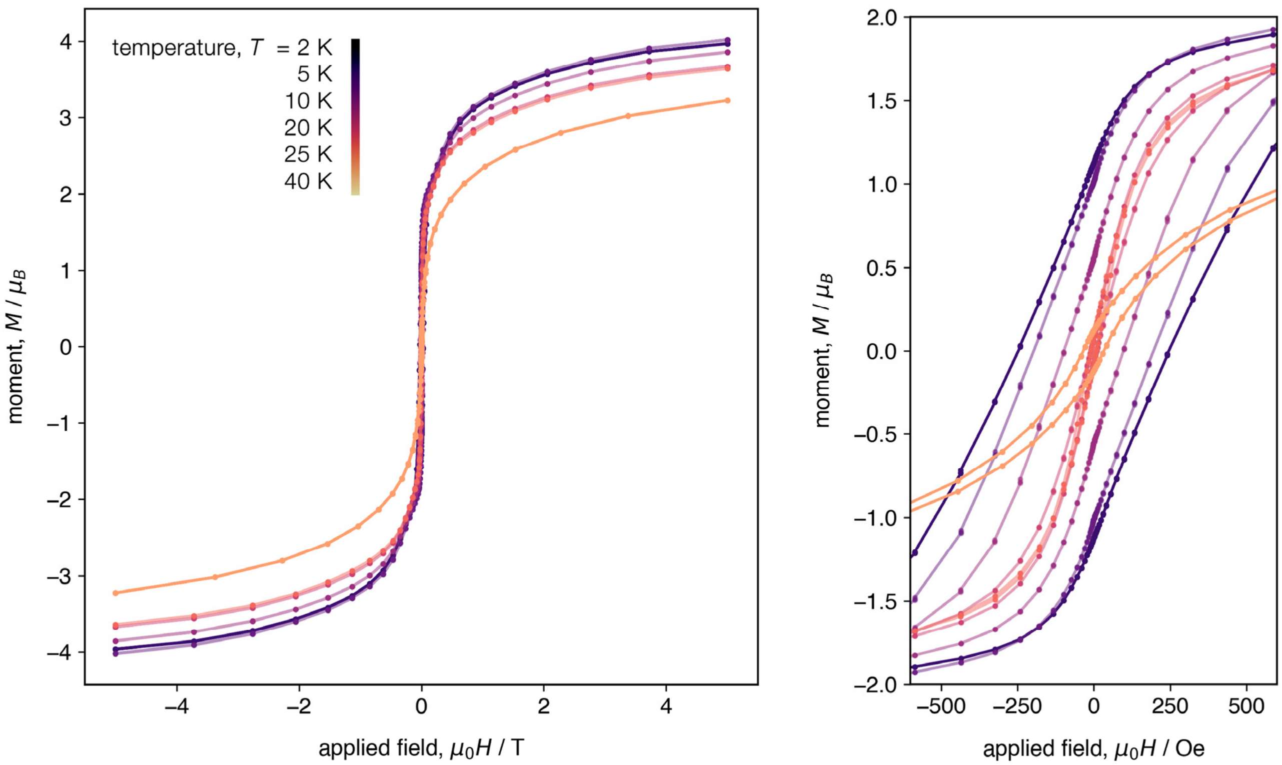

Figure S13. Isothermal DC variable-field magnetization measurements of polycrystalline $(BA)_2CrCl_4$, measured upon cooling from T = 40 K; the region around zero applied field (highlighting increasing remanent magnetization below T_C = 39 K) is enlarged on the right.

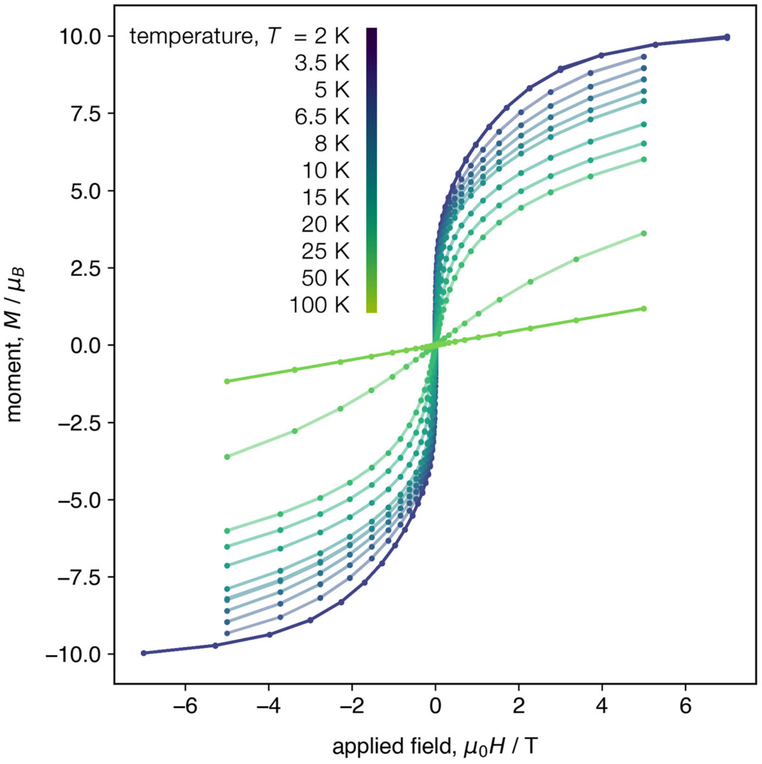

Figure S14. Isothermal DC variable-field magnetization measurements of polycrystalline **$(Ag^{I}Cr^{III})Cr^{II}_2$**, measured upon cooling from T = 100 K.

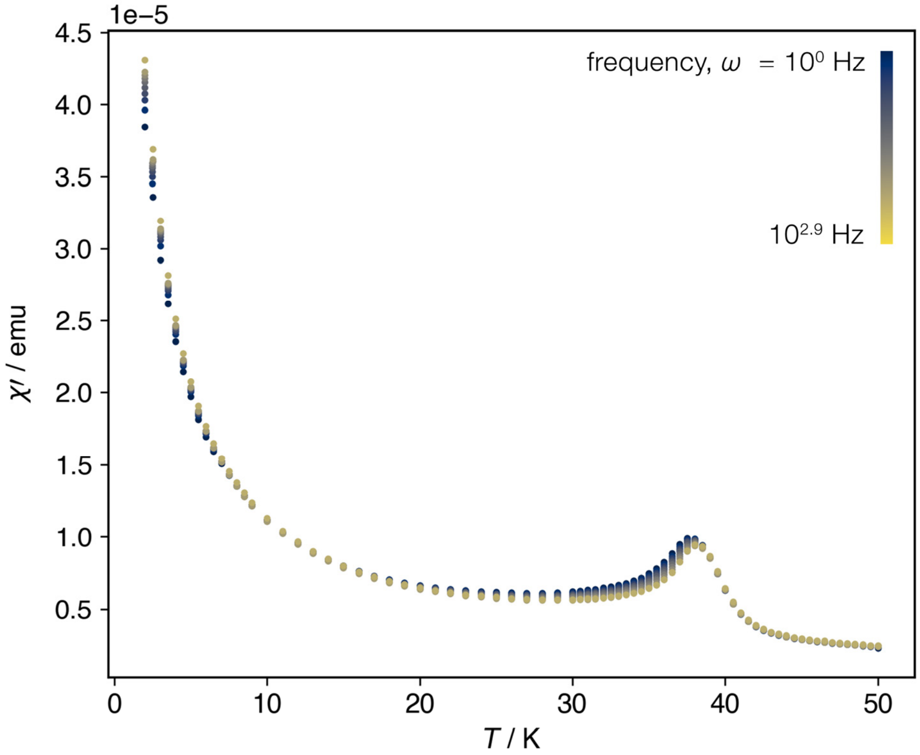

Figure S15. In-phase component of the zero-field AC magnetic susceptibility of $(BA)_2CrCl_4$ crystals after ball milling (following conditions from the synthesis of the mosaic alloys, see Methods), measured with a drive field of 1 Oe and frequency in the range 1 Hz < ω < 775 Hz.

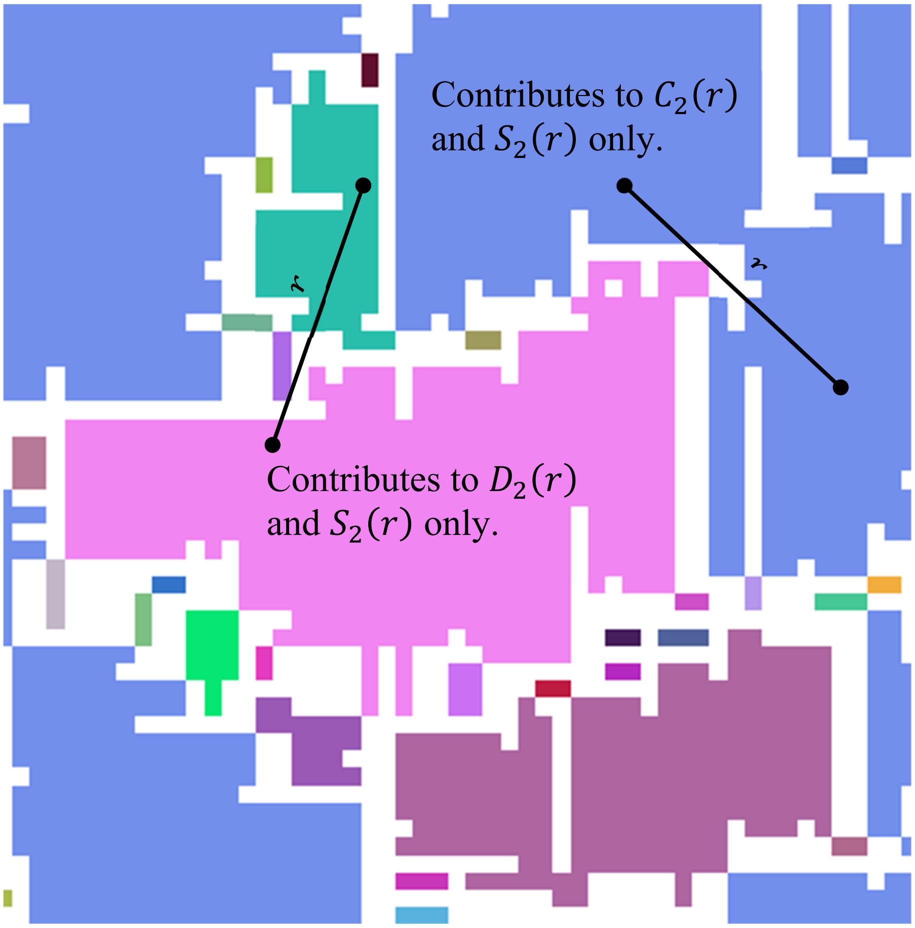

Figure S16. A schematic depicting events that contribute to the two-point *correlation* function $S_2(r)$, two-point *clustering* function $C_2(r)$, as well as the two-point *blocking* function $D_2(r)$. In this image, topologically disconnected Cr^{II} ferromagnetic domains are highlighted in different colors. Individual Cr^{II}–Cl rhombi are represented here by square pixels that combine to form a domain (represented in the same color block) if two or more rhombi are connected. As discussed in the main text, these functions are related by $S_2(r) = C_2(r) + D_2(r)$.[5] The end-points of the length-r line segment on the left-hand side land in two different domains (pink and teal), and thus this event only contributes to $D_2(r)$ and $S_2(r)$. In contrast, the end-points of the length-r line segment on the right-hand side land in the same domain (blue), and thus this event only contributes to $C_2(r)$ and $S_2(r)$.

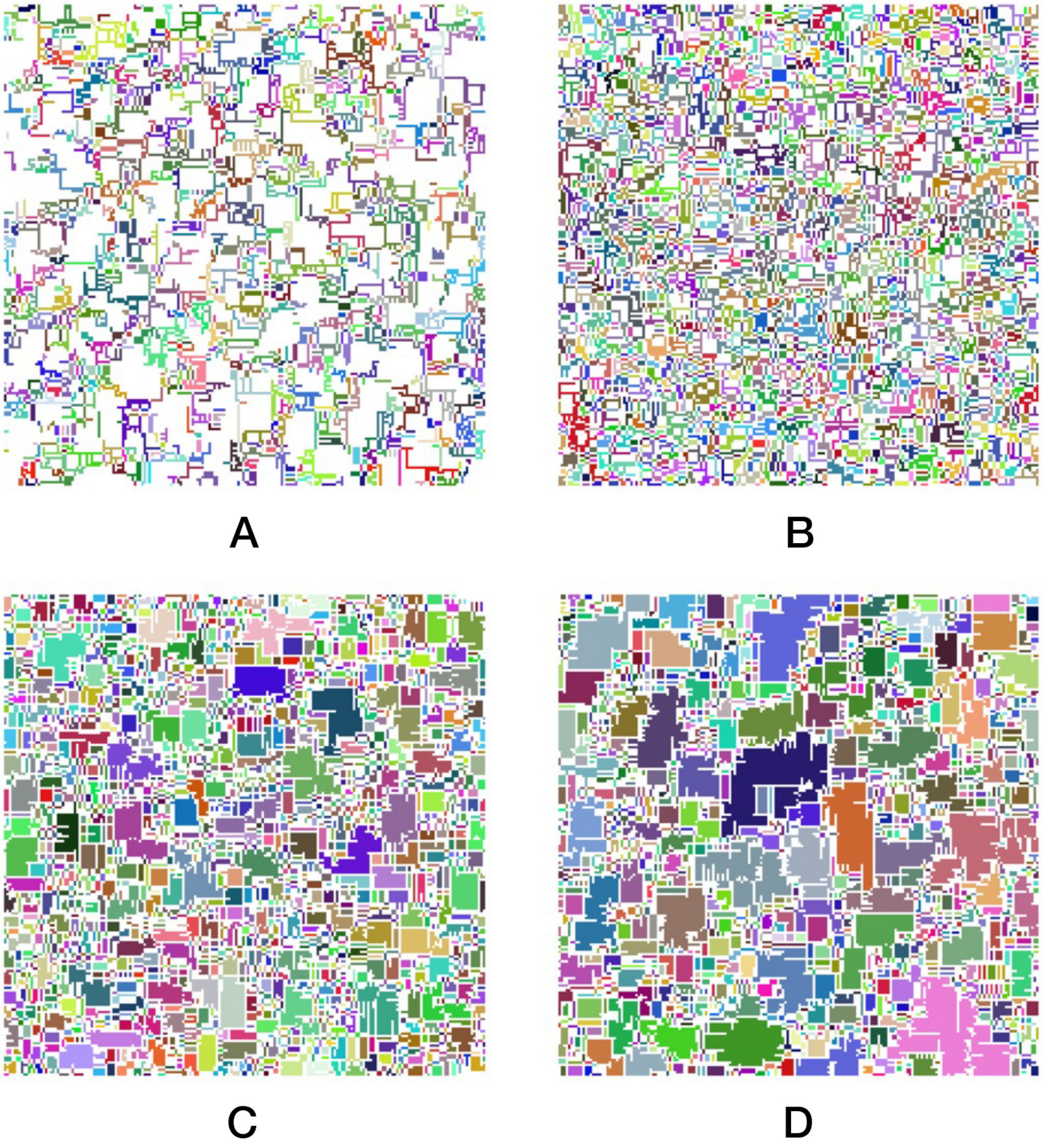

Figure S17. Topologically disconnected ferromagnetic domains identified in LASC structures for mosaic alloys **($Ag^{I}Cr^{III}$)Cr^{II}_{x}** with compositions (**A**) x=1, (**B**) x=2, (**C**) x=3, and (**D**) x=4. These images highlight how the ferromagnetic domains transition from being narrow and branchlike at low Cr^{II} concentrations [(A) and (B)] to wider and more block-like ones as the Cr^{II} concentration increases [(C) and (D)]. In all images, individual grains are highlighted in random colors to assist in visualization, and white space consists of Ag^{I}, Cr^{III}, and individual Cr^{II} not included in domains. The area shown in all images represents the entire 200×200 simulation cell.

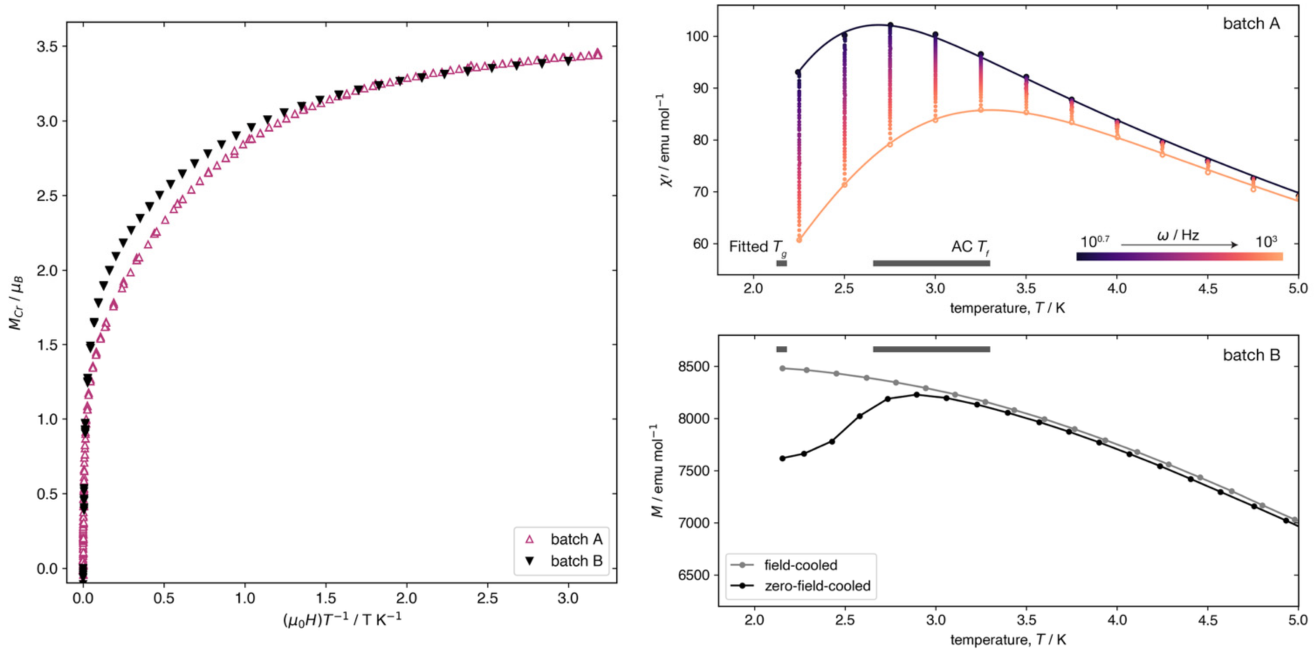

Figure S18. (Left) Reduced isothermal magnetization of **(Ag^ICr^{III})Cr^{II}_2**, demonstrating the approach to saturation at ca. 3.5μ_B, within 5% of the expected value representative of the formula unit with one Cr^{III} and two Cr^{II} ions. (Right) Low-temperature signatures of the spin-glass transition in the frequency-dependent in-phase AC susceptibility (χ', top) and in the DC moment (M, bottom). A comparison of the DC moment under field-cooled and zero-field-cooled conditions shows a divergence (right bottom) in the temperature range associated with spin-glass freezing (right top; see main text, Figure 5C and the associated discussion); the apparent freezing temperature (T_f) from the χ' peak and the fitted transition temperature (T_g) are shown to guide the eye. Here, **batch A** refers to one synthetic batch and data that are reported in the main text (see Figure 5) and **batch B** refers to a different batch, synthesized to demonstrate reproducibility.

Supplementary Tables

Table S1. Crystallographic data for $(BA)_2CrCl_4$ from the Rietveld refinement of high-resolution synchrotron powder X-ray diffraction (see Figure S1).

Compound	$(BA)_2CrCl_4$
Empirical Formula	$C_8Cl_4CrN_2$
Formula Weight, g mol^{-1}	317.91
Temperature, K	300
Crystal System	Monoclinic
Space Group	$P2_1/c$
a, Å	15.9119(7)
b, Å	7.48792(6)
c, Å	7.35611(18)
α, °	90
β, °	103.02(6)
γ, °	90
Volume, Å^3	853.93(21)
Z	2
Density (calculated), g cm^{-3}	1.236
Radiation	Synchrotron (λ = 0.7314 Å)
2θ range, degrees	2 to 40
Reflections	730
Final wR	0.05742

Table S2. Crystallographic data for $(BA)_4AgCrCl_8$ from the Rietveld refinement of high-resolution synchrotron powder X-ray diffraction (see Figure S2).

Compound	$(BA)_4AgCrCl_8$
Empirical Formula	$C_{16}AgCl_8CrN_4$
Formula Weight, g mol^{-1}	691.69
Temperature, K	295
Crystal System	Orthorhombic
Space Group	*Cmm2*
a, Å	7.32894(5)
b, Å	30.82335(20)
c, Å	7.56769(6)
α, °	90
β, °	90
γ, °	90
Volume, Å^3	1709.71(2)
Z	2
Density (calculated), g cm^{-3}	1.343
Radiation	Synchrotron (λ = 0.4597 Å)
2θ range, degrees	0.5 to 25
Reflections	467
Final wR	0.1406

Table S3. Curie-Weiss Law fit parameters from variable-temperature DC susceptibility measurements; calculated values of the effective moment ($\mu_{eff,\ calc}$) correspond to the spin-only moment of Cr.

Compound	θ_{CW} / K	C / emu K mol^{-1}	$\mu_{eff,\ calc}$ / μ_B	$\mu_{eff,\ meas}$ / μ_B
$(BA)_2CrCl_4$	64.25	3.02	4.90	4.92
$(BA)_4AgCrCl_8$	–0.63	1.86	3.87	3.86

Table S4. Inductively coupled plasma–optical emission spectroscopy measurements of the relative composition of Ag and Cr from digested **$(Ag^{I}Cr^{III})Cr^{II}_2$** powder (see the Methods section, main text); n is the number of independent measurements (or combined, in the case of the pooled statistics).

Sample	n	Cr:Ag (mol:mol)
Stock	3	2.89(2)
Dilution (1:2)	3	2.94(1)
Dilution (1:10)	3	2.87(1)
Pooled	9	2.90(1)

Supplementary References

1. Skolnick, M. & Torquato, S. Communication: Modeling layered mosaic perovskite alloy microstructures across length scales via a packing algorithm. *J. Chem. Phys.* **163**, 201101 (2025).
2. Toby, B. H. & Von Dreele, R. B. GSAS-II: the genesis of a modern open-source all purpose crystallography software package. *J. Appl. Crystallogr.* **46**, 544-549 (2013).
3. Christy, A. A., Kvalheim, O. M. & Velapoldi, R. A. Quantitative analysis in diffuse reflectance spectrometry: A modified Kubelka-Munk equation. *Vibrational Spectroscopy* **9**, 19-27 (1995).
4. Lindquist, K. P., Vigil, J. A., Su, A. C. & Karunadasa, H. I. in *Comprehensive Inorganic Chemistry III,* Vol. 44.15 - A practical guide to Three-dimensional halide perovskites: Structure, synthesis, and measurement, pp. 499-559 (Elsevier, 2023).
5. Torquato, S., Beasley, J. D. & Chiew, Y. C. Two-point cluster function for continuum percolation. *J. Chem. Phys.* **88**, 6540-6547 (1988).
